# Electronic structure analysis of borylenes and their role in small molecule activation

**DOI:** 10.1039/d6ra00186f

**Published:** 2026-02-20

**Authors:** Vikiho Wotsa, Chinnappan Sivasankar

**Affiliations:** a Catalysis and Energy Laboratory, Department of Chemistry, Pondicherry University (A Central University) Puducherry 605014 India siva.che@pondiuni.ac.in

## Abstract

The activation of small molecules by non-metal-based systems has emerged as a compelling area of study, particularly with respect to main-group element chemistry as an alternative to transition metal catalysis. Here, we investigate the activation of two classes of small molecules by the CAAC-stabilized borylene species CAAC–B–N(SiMe_3_)_2_. Type A molecules include CO, NO^+^, C_2_H_4_, PH_3_, CN^−^, and N_2_, while type B includes CH_4_, C_6_H_6_, and H_2_. A series of computational analyses have been carried out to elucidate the electronic structure and bonding characteristics of the borylene complexes. Energy decomposition analysis and frontier molecular orbital investigation reveal that both types of small molecules engage in significant donor–acceptor interactions with the borylene center. Specifically, in type A complexes, there is a notable donation of electron density from the substrate into the vacant p-orbital of boron, accompanied by π-back donation from the filled orbitals of the borylene into the acceptor orbitals of the small molecule. In contrast, type B complexes are characterized predominantly by π-backdonation from the borylene into the empty σ* orbitals of the small-molecule substrates. These findings provide an insight into the reactivity of low-valent boron species and underscore their potential in small-molecule activation chemistry.

## Abstract 2

The activation of small molecules by non-metal-based systems has emerged as a compelling area of study, particularly with respect to main-group element chemistry as an alternative to transition metal catalysis.

## Graphical abstract

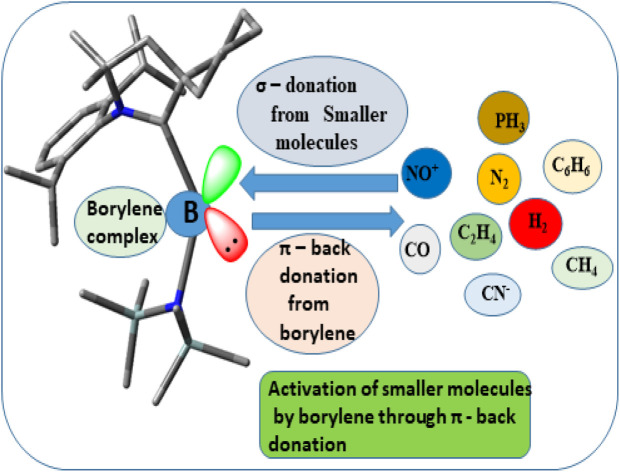

## 1. Introduction

The activation of small molecules represents a rapidly advancing frontier in 21st-century chemical research, particularly as efforts intensify to develop sustainable and metal-free alternatives to traditional transition-metal-based catalysis. Small molecules such as nitric oxide (NO), carbon monoxide (CO), and methane (CH_4_) are of exceptional industrial, environmental, and biological importance, with applications spanning from materials science to pharmaceutical chemistry. Nitric oxide (NO), for example, plays a central role in the chemical industry as a precursor to nitric acid, a vital reagent for fertilizers and explosives.^1,2^ Carbon monoxide (CO), identified as a discrete molecule, has since been revealed to possess both toxic and therapeutic properties. In mammalian systems, CO is endogenously generated by heme oxygenases and exerts a variety of physiological effects.^3–6^ Industrially, CO is a core component of syngas and serves as a crucial feedstock for the synthesis of methanol, acetic acid, and liquid hydrocarbons through processes such as Fischer–Tropsch synthesis and hydroformylation.^7^ Hydrocarbons such as methane and ethylene are similarly vital to modern chemical economies. Methane, a major constituent of natural gas, is utilized in steam methane reforming to produce hydrogen and methanol and considered a promising transitional fuel in the movement toward cleaner energy.^8–10^ Ethylene, one of the most important petrochemical feedstocks, serves as a precursor for a wide array of industrial chemicals and polymers.^11^

Additionally, hydrogen (H_2_) has emerged as both an indispensable industrial feedstock and a clean energy carrier. Its roles include hydro processing in oil refineries, ammonia synthesis, and powering fuel cells with water as the sole by product.^12,13^ Phosphine (PH_3_) serves as a precursor for the synthesis of organophosphorus compounds and, more recently, has shown potential in medicinal chemistry through gold-based phosphine complexes with antitumor activity.^14,15^ Cyanide (CN^−^) ions have long been utilized in metallurgy, and the production of polymers and pesticides.^16^ Dinitrogen (N_2_), a diatomic molecule is central to both industrial and biological nitrogen fixation, forming the cornerstone of global nitrogen cycling and ammonia production.^17,18^

Despite major advances in transition-metal catalysis for small-molecule activation, including Pd-catalysed oxidative coupling of methane to acetic acid *via* C–H activation and carbonylation,^19^ hopcalite (CuMnO_*x*_) for low-temperature CO oxidation,^20,21^ heterobimetallic MFe(CO)_4_^−^ clusters (M = Ti, V, Cr),^22^ and cobalt oxide clusters active in CO oxidation,^23,24^ the development of metal-free systems capable of activating inert molecules such as N_2_, CO, H_2_, and PH_3_ remains a significant challenge and opportunity.

In this context, low-valent boron species particularly borylenes have emerged as promising transition-metal mimics due to their ability to accept electron density and engage in π-back-donation. A seminal contribution by Braunschweig *et al.*^25^ reported the first stable terminal borylene complex, [(OC)_5_WBN(SiMe_3_)_2_], establishing a foundation for main-group small-molecule activation. Bertrand and co-workers introduced neutral tricoordinate organoboranes isoelectronic with amines, which undergo one-electron oxidation to radical cations; *ab initio* studies showed that the HOMO and SOMO are localized on the boron p orbital, consistent with borylene and borinylium character.^26^

Metal-free activation of H_2_ was first demonstrated by Stephan *et al.* using phosphine/borane frustrated Lewis pairs (FLPs), where reversible H_2_ cleavage and heterolytic splitting at room temperature yielded phosphonium/borohydride salts with catalytic relevance.^27,28^ Subsequent breakthroughs include the isolation of a stable B–B triple bond,^29^ FLP-mediated heterolytic H_2_ cleavage using carbene–borane systems,^30^ and H_2_ activation by *t*Bu_3_P/B(C_6_F_5_)_3_, forming [*t*Bu_3_PH]^+^[HB(C_6_F_5_)_3_]^−^ alongside phosphinoborane products.^31^

The first evidence of boride borylene ligand tautomerism in a rhodium complex was reported recently, where the intramolecular C–C bond formation within the ligand backbone, producing a boryl complex which reveals how ligand reactivity and tautomerism can dictate the outcome in low-valent boron coordination chemistry.^32^ The activation of elemental sulfur (S_8_) by a borafluorene derivative, leading to the isolation of boryl-linked S_7_ and S_8_ acyclic sulfur chains (catenates) highlights how electron-rich boron centers can mediate challenging S–S bond activation and stabilise unusual sulfur-chain motifs.^33^ Furthermore, the boron-centered reductive elimination was performed *via* arene extrusion from halogenated boranorbornadienes.^34^

**Fig. 1:**
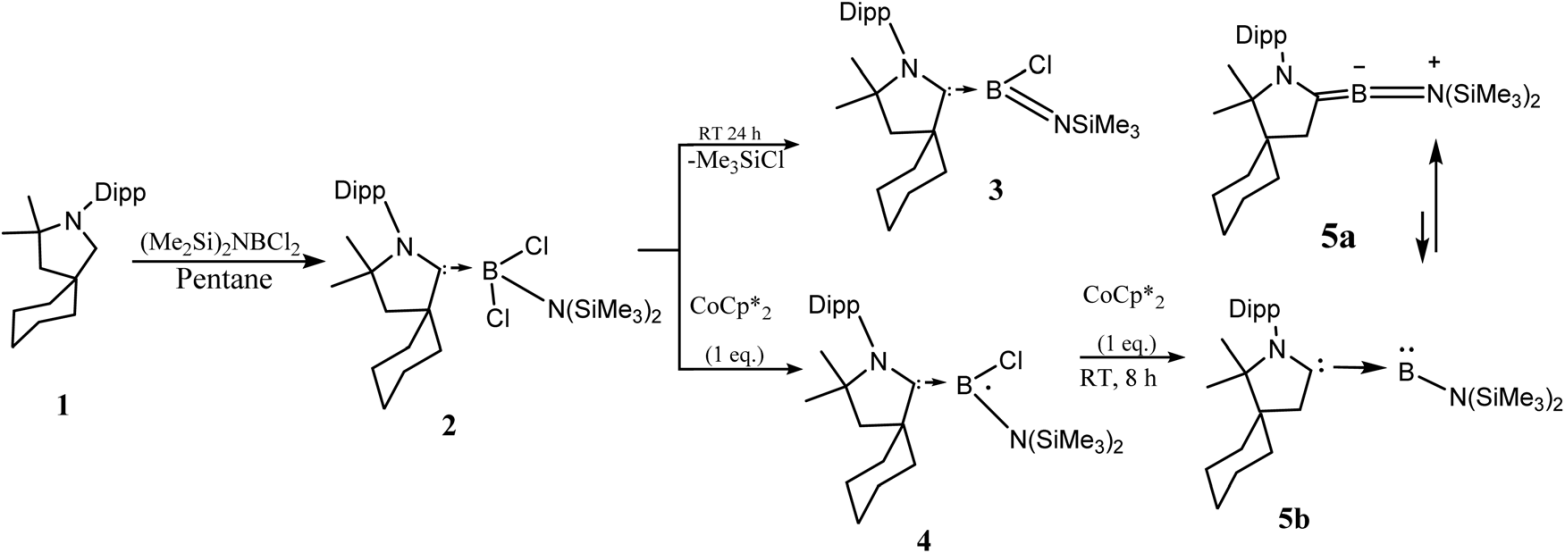
Bertrand's schematic representation of the synthesis of CAAC–B–N(SiMe_3_)_2_ (5a). Reproduced or adapted with permission from ref. 35.

Bertrand *et al.*,^35^ reported the synthesis of a CAAC-stabilized aminoborylene, a low-valent boron species that is isoelectronic with singlet carbenes and displays carbene-like electronic structure and reactivity. Both an aminoboryl radical and the corresponding borylene adduct were isolated, which can activate small molecules such as CO and H_2_, highlighting that appropriately stabilized boron centers can mimic classic carbene behavior and expanding the scope of reactive main-group compounds (Fig. 1).

The activation of CO through ketenyl by PPh_3_/CO exchange provides a new strategy of utilizing an exchange reaction of a phosphine with carbon monoxide at a metalated ylide gives rise to a series of novel ketenyl anions.^36^ In this present study, we have investigated the reactivity of the Bertrand-type borylene compound, [CAAC–B–N(SiMe_3_)_2_], toward a series of small molecules, including benzene (C_6_H_6_), methane (CH_4_), ethylene (C_2_H_4_), nitrosonium (NO^+^), cyanide (CN^−^), dinitrogen (N_2_), dihydrogen (H_2_), carbon monoxide (CO), and phosphine (PH_3_). This work places significant emphasis on understanding the role of the σ-donating and π-back bonding characteristics of the borylene fragment in facilitating small-molecule activation. To gain mechanistic insights, density functional theory (DFT) calculations were performed, and the computational results are presented herein.

## 2. Computational methods

Geometry optimizations for complexes 1–9 were carried out using the M06-2X^37^ functional with the 6-311G**^38^ basis set for all atoms, without the inclusion of additional dispersion corrections. The M06-2X functional exhibits high accuracy in describing main-group thermochemistry, noncovalent interactions, and barrier heights. The dispersion interactions are often important for weakly bound systems such as benzene and methane complexes however, M06-2X incorporates empirical parameterization which partially accounts for the medium range dispersion effects, making it suitable for capturing both covalent and noncovalent contributions without the need for applying an explicit dispersion correction.^37,39^ At the same level of theory and basis set, we have adopted natural bond orbital analysis (NBO)^40^ to calculate the natural population analysis (NPA), Wiberg bond index (WBI)^41^ calculations, molecular orbital analysis, and electrostatic potential mapping to study the bonding behaviour of electrons in these complexes. All these calculations were executed using the Gaussian 16 software package.^42^ Energy decomposition analysis (EDA)^43^ was performed at the generalized gradient approximation (GGA) level using the Becke–Perdew (BP86)^44,45^ exchange–correlation functional in combination with a triple-zeta basis set including double polarization functions (TZ2P).^46–48^ These calculations were carried out using the Amsterdam Density Functional (ADF 2017)^43^ software package, employing the geometries optimized at the M06-2X/6-311G** level. In the energy decomposition analysis (EDA), the choices of fragments for each complex (NCB–X) was partitioned into two fragments; the borylene (NCB) moiety and (X) the coordinated ligand. This approach allows the decomposition to quantify the physical contributions such as electrostatic, Pauli repulsion, and orbital interactions which arises from the donor–acceptor interaction between the boron center and the ligand. Geometry optimizations were performed using the hybrid meta-GGA M06-2X functional with the 6-311G** basis set, as this level of theory has been shown to provide reliable equilibrium geometries for main-group and transition-metal complexes.^37^ Energy decomposition analysis (EDA) was carried out at the BP86/TZ2P level, which is the standard and well-validated framework for EDA implementations and allows for a consistent and physically meaningful partitioning of the interaction energy.^49^ The Cartesian coordinates of the complete complexes are provided in the SI.

## 3. Results and discussion

### 3.1. Optimized geometries and bonding analysis

**Fig. 2:**
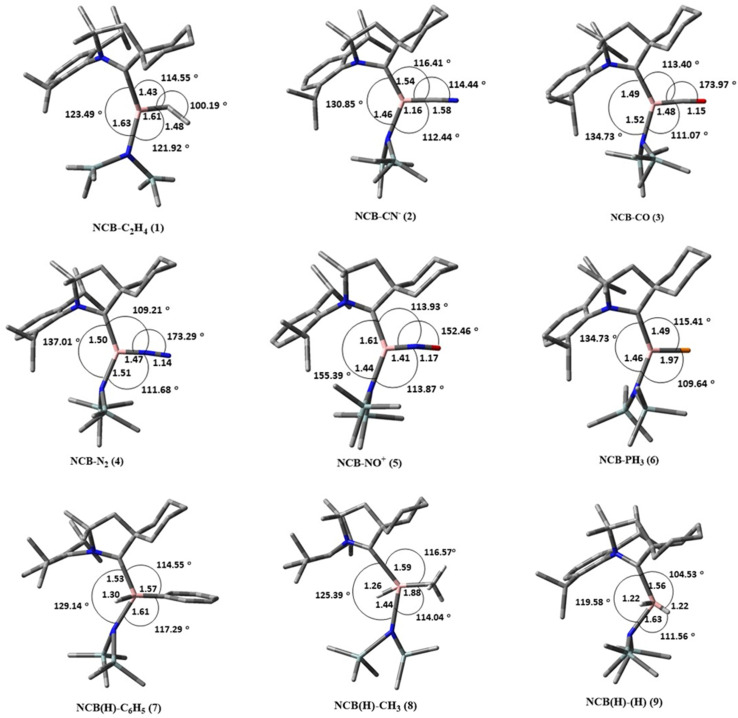
Optimized geometries of the complex 1–6 (type-A complex) and 7–9 (type-B complex) studied in this work. Calculated bond length in Å and bond angle in °. Hydrogen atoms were removed for clarity.

The calculated geometries of complexes 1–9 (NCB–X) are shown in Fig. 2, where NCB = CAAC–B–N(SiMe_3_)_2_ and X = C_2_H_4_ (1), CN^−^ (2), CO (3), N_2_ (4), NO^+^ (5), PH_3_ (6), C_6_H_6_ (7), CH_4_ (8), H_2_ (9), with the selected bond lengths and bond angles. The experimental solid state structure of the CAAC–B–N(SiMe_3_)_2_ borylene complex [Fig. 1 (5a)]^35^ shows a B–C(CAAC) bond length of 1.401(5) Å and a B–N bond length of 1.382(3) Å, with the boron centre adopting almost a linear geometry N–C–B angle = 174.8(3)°. DFT optimized structure reproduces these geometrical features, with calculated B–N and B–C (CAAC) bond lengths of 1.38 Å and 1.41 Å, respectively and a bond angle (N–C–B) of 175.01°.^40^

**Fig. 3:**
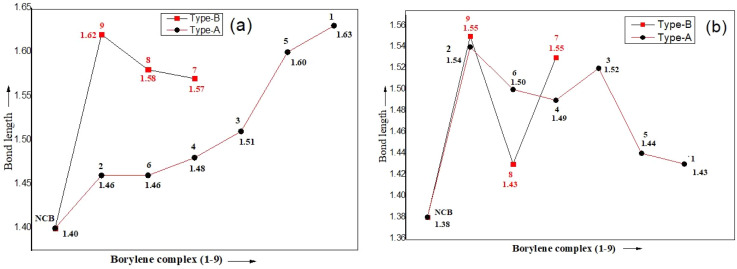
Plot of bond length in borylene complex 1–9. (a) Bond length (Å) of B–L1 (B–CAAC) and (b) bond length (Å) of B–L2 [B–N(SiMe_3_)_2_].

Upon activation of various small molecules (1–9), significant changes in both geometry and electronic structure were observed. The characteristic B=N and B=C(CAAC) double-bond character of the parent borylene were diminished, giving rise to longer B–N and B–C single bonds in the resulting adducts. These bond length elongations reflect the reduction in π-bonding character upon interaction with the small molecules, and the extent of this change varies depending on the nature of the incoming substrate. Moreover, significant variations were observed in the bond lengths, bond angles, and charge distribution around the borylene center (Table S2). The calculated trend for the bond lengths of the borylene with different adducts are as follows, in the type A complex; NCB–PH_3_ < NCB–C_2_H_4_ < NCB–CN^−^ < NCB–CO < NCB–N_2_ < NCB–NO^+^ and in type B complex; NCB(H)–CH_3_ < NCB(H)–C_6_H_5_ < NCB(H)–(H). The elongation of the B–C(CAAC) and B–N bonds relative to the parent borylene reflects the partial loss of the B=C and B=N multiple bond character due to coordination (Fig. 3). Complex 1 (ethylene adduct) exhibits a B–C bond length of 1.61 Å and a N–C–B bond angle of 123.49°, which is an obtuse angle. This distortion can be attributed to the presence of π-bonds of ethylene, which perturbs the electron density at boron and induces pyramidalization. Similar trends are seen in complexes 7 and 8 (benzene and methane adducts), with B–C bond lengths of 1.57–1.58 Å and N–C–B angles in the range of 125–129°.

Type-B complexes, including NCB(H)–C_6_H_5_, NCB(H)–CH_3_, and NCB(H)(H), exhibit negligible π-acceptor character which is evident form the longer B–L1 and B–L2 bond lengths in comparison with the other complexes. As shown in Table S2 (SI), the bond lengths increase from the experimental values of 1.401(5) Å (B–L1) and 1.382(5) Å (B–L2) in uncoordinated N–C–B to 1.57 Å, 1.58 Å (B–L1) and 1.53 Å (B–L2) in NCB(H)–C_6_H_5_. A similar trend were observed in the NCB(H)–CH_3_ and NCB(H)(H) complexes. The optimized geometry in NCB(H)–CH_3_ reveals a bent N–C–B bond angle of 130.4°, with hydrogen abstraction from the methane leading to the formation of a new B–H bond.

In contrast, type A complexes show relatively smaller increases in bond lengths. Adducts such as NCB–CO and NCB–NO^+^ which possess significant π-acceptor character, resulting in stronger backbonding and shorter B–L2 bond lengths. For example, the B–L2 bond length is 1.50 Å in NCB–CO and 1.44 Å in NCB–NO^+^. Complexes with isoelectronic ligands namely, CN^−^, N_2_ and CO exhibit obtuse geometries, with N–C–B bond angles approaching 130–137°, indicative of sp^2^-hybridized boron centers. In the case of NCB–PH_3_, a comparatively smaller shortening of bond length B–P (1.91 Å) was observed relative to the standard B–P bond length of (1.96 Å).

These interactions preserve substantial charge donations from the borylene fragment, stabilizing the linear arrangements. Bickelhaupt *et al.*,^50^ demonstrated that CO bonding is governed by 2s–2p orbital mixing rather than electronegativity differences. Strong mixing on carbon inverts the σ and π orbital ordering, polarizing the 5σ orbital toward carbon and accounting for CO's small dipole moment and its preference for carbon-end coordination in metal complexes. The activation of CO using the B_2_(NHC)_2_ which has no d orbitals indicated that the activation occurred due to HOMO–LUMO swaps, the π* orbitals of CO are able to accept electrons from B_2_(NHC)_2_, and the significant electron back donation eventually weakens and activates the CO triple.^51^ Interestingly, the NO^+^ complex (8) shows a slightly bent B–N–O geometry (152.46°), likely due to the asymmetric charge distribution in the NO^+^ fragment. The computed bond distances between the boron atom and the sp^2^-hybridized carbon and nitrogen atoms in CO, N_2_, and NO^+^ are relatively consistent, ranging from 1.42 to 1.48 Å.

The vibrational frequency of the complex NCB–C_2_H_4_ (1) appear at 1070 cm^−1^ for the C_α_–C_β_ bond, and 3160 cm^−1^ for the C_α_–H stretching mode (Table 1). In comparison, free C_2_H_4_ exhibits characteristic vibrational bands at 1623 cm^−1^ for the C–C stretch and 3100 cm^−1^ for the C–H stretch. The significant red shift observed in the C_α_–C_β_ stretching frequency upon complex formation indicates a weakening of the C–C bond arises due to coordination of NCB fragment with adduct. In the complexes such as; NCB–CN^−^, NCB–CO, NCB–N_2_, NCB–NO^+^, and NCB–PH_3_, new B–X (X = C, N, P) bonding interactions are supported by the vibrational bands in the range of 777–1097 cm^−1^. The stretching frequencies of the ligands shows a substantial shifts compared to their free states stretching frequencies after the coordination with the NCB fragment. For example, the C–O stretching frequency in CO shifts from 2134 cm^−1^ in free state to 2089 cm^−1^ in complex. Similarly, N_2_ and NO^+^ exhibit significant red shifts from 2331 to 2144 cm^−1^ and from 2376 to 1879 cm^−1^, respectively. In contrast, the P–H stretching frequency in PH_3_ shifts from 2321 cm^−1^ in the free molecule to 2399 cm^−1^ upon coordination, reflecting altered bonding interactions with the NCB fragment.

**Table 1:** Calculated vibrational frequency of complex 1–9. For atom indications refer SI Fig. S1

System	Vibrational frequency (cm^−1^)				
	(In complex)			(Free state)	
NCB–C_2_H_4_ (1)	1053 (B–C_α_)	1070 (C_α_–C_β_)	3160 (C_α_–H)	1623 (C–C)	3100 (C–H)
NCB–CN^−^ (2)	1034 (B–C_α_)	2273 (C_α_–N)	—	2250 (C–N)	—
NCB–CO (3)	1063 (B–C_α_)	2089 (C_α_–O)	—	2134 (C–O)	—
NCB–N_2_ (4)	1002 (B–N_α_)	2144 (N_α_–N_β_)	—	2331 (N–N)	—
NCB–NO^+^ (5)	1097 (B–N_α_)	1879 (N_α_–O)	—	2376 (N–O)	—
NCB–PH_3_ (6)	777 (B–P)	2399 (P–H)	—	2321 (P–H)	—
NCB(H)–C_6_H_5_ (7)	1204 (B–C_α_)	2066 (B–H)	—	3047 (C–H)	—
NCB(H)–CH_3_ (8)	1273 (B–C_α_)	2325 (B–H)	—	2917 (C–H)	—
NCB(H)–(H) (9)	2419 (B–H_α_)	2407 (B–H_β_)	—	4400 (H–H)	—

In the case of NCB(H)–C_6_H_5_ and NCB(H)–CH_3_ complexes, B–C_α_ stretching vibrations are observed at 1204 and 1273 cm^−1^, respectively, along with terminal B–H stretching frequencies at 2066 cm^−1^ for NCB(H)–C_6_H_5_ and 2325 cm^−1^ for NCB(H)–CH_3_. In comparison, the free C–H stretching frequencies of C_6_H_5_ and CH_3_ appear at 3047 and 2917 cm^−1^, respectively. In the case of NCB(H)–(H) complex, as evident two B–H modes of stretching frequencies are observed at 2419 and 2407 cm^−1^, which are significantly oriented towards the red shift relative to the H–H stretching frequency in free state at 4400 cm^−1^, indicating a substantial weakening of the H–H bond upon the coordination with NCB fragments.

The NPA (natural population analysis) shows that the boron center bears a negative charge in all the complexes (see SI Table S3), which indicates its strong electron-accepting characteristics at the boron centre. Of all the complexes, the most negative charge is observed in the ethylene complex NCB–C_2_H_4_ (1) −1.036 (see SI Fig. S1 for the subscript indication on the atoms), consistent with significant electron donation from the substrate. In contrast, the boron center in the NCB(H)–(H) complex is almost neutral (−0.005), which indicates the weak donor–acceptor interaction in the complex. The bond order and Wiberg bond index (WBI) analyses (SI, Table S4) provide insights into the electronic structure of the NCB and small molecule complexes. In general, B–X bonds (X = C, N, P, H) exhibit single-bond character, with WBIs ranging from 0.49 to 1.16, reflecting variable electron sharing with the substrate. For example, the B–C bond in NCB–C_2_H_4_ and NCB(H)–C_6_H_5_ shows moderate Wiberg values of 0.82 to 0.84, which indicates the significant covalent interaction, whereas B–H bonds in NCB(H)–CH_3_ and NCB(H)–(H) have slightly lower values 0.52–0.93, which is an evident of weaker orbital overlap. The C–N triple bond in NCB–CN^−^ exhibits a very high Wiberg value of 2.82, reflecting a strong electron delocalization and a multiple-bond character, whereas the C–O bond in NCB–CO shows a almost or near zero Wiberg value of −0.01. This indicates the weak overlap and significant polarization between the boron center and CO adduct. In the case of B–N bond in NCB–N_2_ and NCB–NO^+^, the Wiberg value of 0.90 and 1.16 reflects with their strong σ-donation and partial π-back bonding characteristics. Moreover, the B–P bond in NCB–PH_3_ also has a Wiberg value of 0.90, highlighting its longer bond length and weaker interaction. From the above results it can be drawn that, while Wiberg bond indices and bond orders provide useful qualitative insight into electron sharing and covalent bond characteristics, however the extreme or the negative values should be interpreted cautiously, as they may also reflect the delocalization, polarization, or limitations of NBO analysis rather than classical bond strength.^40,41,52,53^

**Fig. 4:**
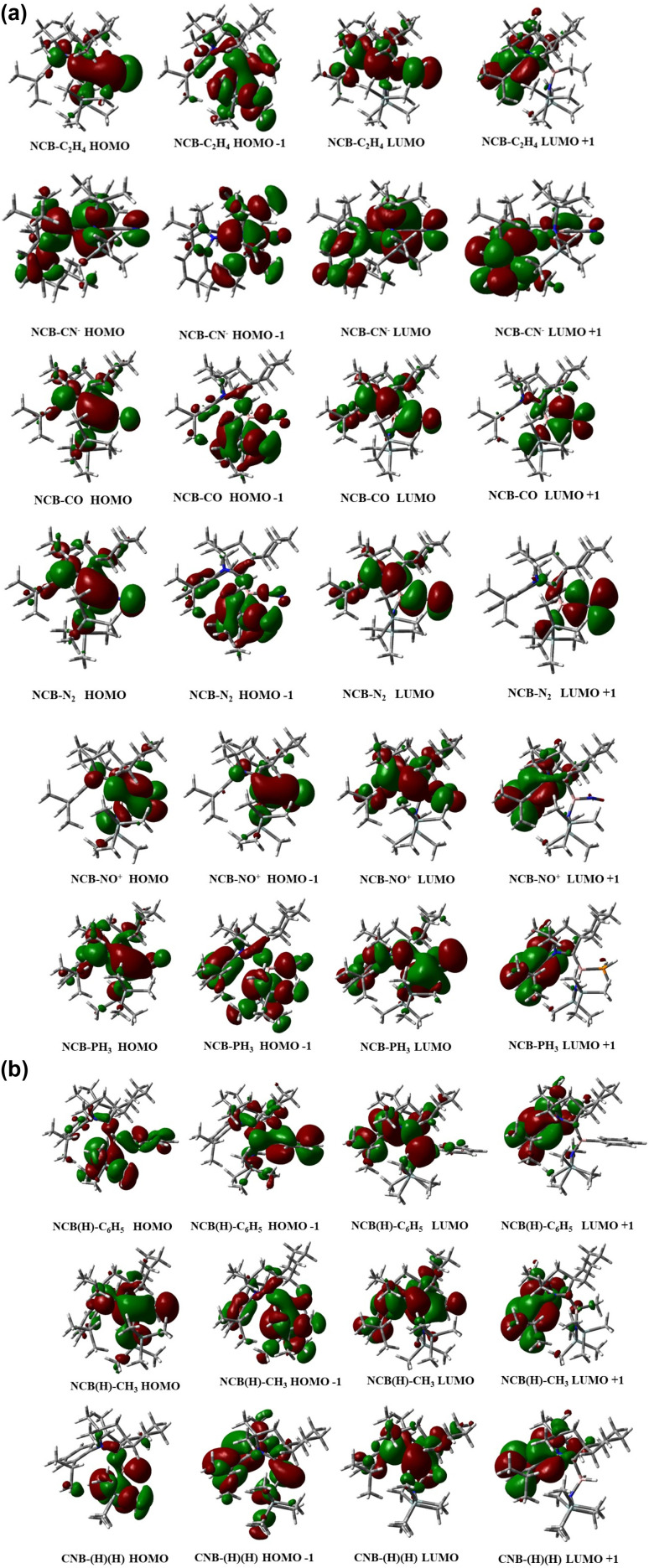
(a) Orbital involved in the HOMO and LUMO of complex 1–6. (b) Orbital involved in the HOMO and LUMO of complex 7–9.

**Fig. 5:**
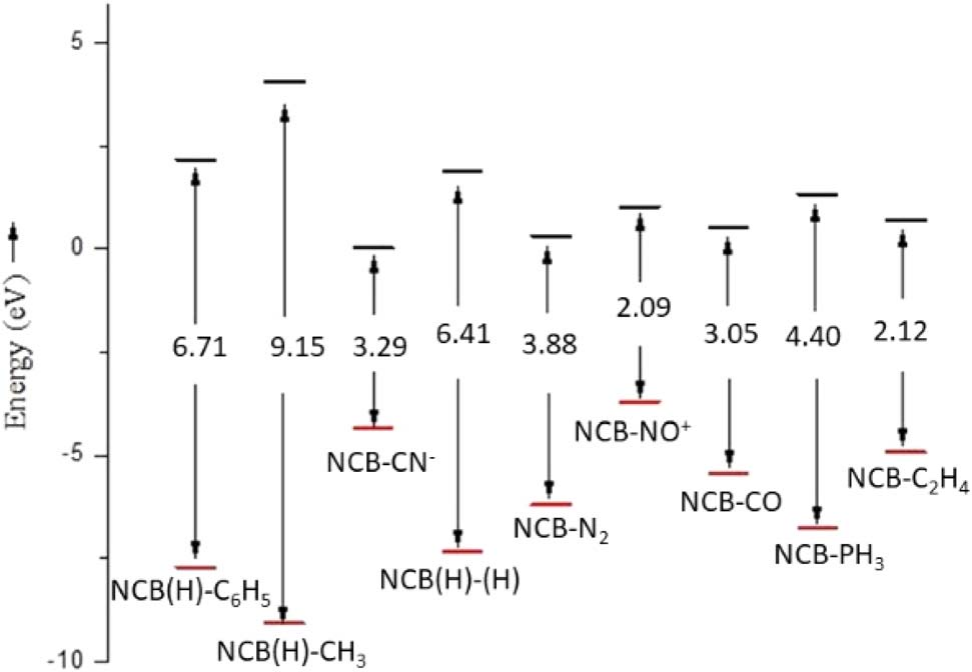
Orbital involved in the HOMO and LUMO of complex 1–9 (energy gap in eV).

The frontier molecular orbitals analysis of the complex 1–9 are shown in Fig. 4a and b, and their corresponding HOMO–LUMO energy gaps are summarized in Fig. 5. Among the complexes, 8 (NO^+^) exhibits the smallest HOMO–LUMO gap with 2.09 eV. In contrast, complex 5 (CO) shows a larger energy gap of 3.05 eV, which reflects a comparatively more electronically stabilized system. The FMO analysis reveals that in the complex 3, 4 and 5, the highest occupied molecular orbital (HOMO) involves a three centre bonding interaction between the boron atom and the coordinated adducts CO, N_2_ and NO^+^. This interaction arises from donation of electron density from the filled orbital of boron into the π* orbitals of the corresponding adducts, which is closely resembling with the transition-metal complexes.

The isoelectronic complexes 2 (CN^−^) and 4 (N_2_) display HOMO–LUMO gaps of 3.29 eV and 3.88 eV respectively, reflecting increased electronic stabilization arising from strong donor–acceptor interactions. Complex 6 (PH_3_) has an energy gap of 4.40 eV falling between the extremes observed across the series of all the other complexes.

Although boron does not possess two classical lone pairs, the frontier molecular orbitals include an occupied σ-type orbital and a higher-energy π-type orbital with significant boron character, reflecting the electronic structure of the borylene centre. The B–P interaction in NCB–PH_3_ arises primarily from donation of the phosphorus lone pair into the vacant orbitals of the boron centre, consistent with a donor–acceptor bonding description.

**Fig. 6:**
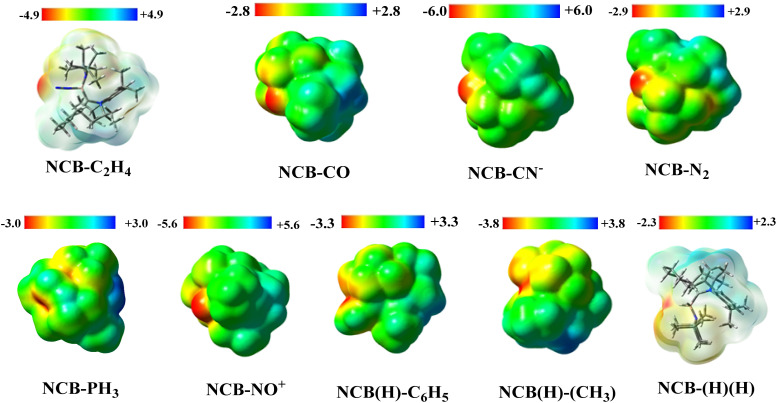
Plot of the ESP electrostatic potentials of complex 1–9. ESP is mapped on the 0.001 au an electron density isosurfaces, in the range −2.3 to −6.0 au (CNB–C_2_H_4_ and CNB–(H)(H)) in skeletal overlay to show the orientation of the bonds.

The molecular electrostatic potential surface maps are shown in Fig. 6, which provides a complementary insight into the electronic environments of the borylene and small molecules adduct. Among the series, the CN^−^ adduct exhibits the most negative potential of −6.0, reflecting strong electron donation to the boron centre, consistent with its high σ and π donor characters. Complex 5 (NO^+^) with −5.6 and 1 (C_2_H_4_) −4.9, also displays significant negative ESP, which reflects the significant orbital overlap and electron density accumulation at the boron centre.

In contrast, weaker donor adducts such as, PH_3_ (−3.0), C_6_H_6_ (−3.3), CH_4_ (−3.8) and H_2_ (−2.3) shows a progressively less negative potentials, consistent with reduced electron donation and smaller perturbations of the boron electron density. In the case of complex 3 (CO) and 4 (N_2_), despite having the differences in the bonding topology and orbital interactions, they both exhibits a comparable electrostatic potential surface of −2.8 and −2.9 respectively. This observation demonstrates that similar charge distributions at the boron centre can arise from distinct donor–acceptor mechanisms.

### 3.2. Energy decomposition analysis

The energy decomposition analysis EDA was employed to investigate quantitatively the bonding nature between the CAAC-stabilized borylene [CAAC–B–N(SiMe_3_)_2_] and the small molecules. The EDA consists of the total instantaneous interaction energy (Δ*E*_int_) between the two interacting fragments is partition into three primary components (which, shown in eqn (I)): the quasi-classical electrostatic interaction (Δ*E*_elstat_), which represents the coulombic attraction between the unperturbed charge distributions of the fragments and is typically attractive. The orbital interaction (Δ*E*_orb_), which accounts for charge transfer, polarization, and electron sharing effects that reflect covalent bonding contributions and the Pauli repulsion (Δ*E*_Pauli_), which describes the destabilizing interaction due to the anti-symmetry requirement of wave functions involving electrons with the same spin (*i.e.*, steric repulsion). This decomposition allows for an intricate understanding of the physical origins of bonding in these systems.^54–60^

I Δ*E*_int_ = Δ*E*_elect_ + Δ*E*_orb_ + Δ*E*_Pauli_

**Fig. 7:**
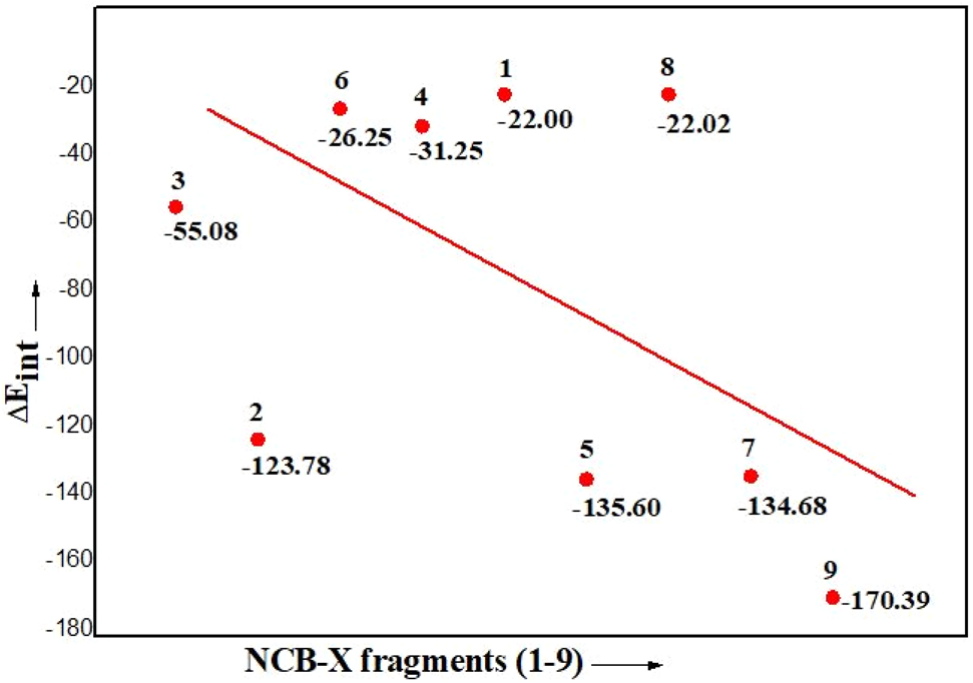
Plot of Δ*E*_int_ (kcal mol^−1^) of borylene complex 1–9.

The results of the EDA calculations for complex 1–9 are summarize in Table 2 and the Δ*E*_int_ plot of complex 1–9 is given in Fig. 7. The borylene fragment was treated as the acceptor, while the small molecules were considered as donor fragments. The orbital interaction component (Δ*E*_orb_) serves as an indicator of the covalent character of the NCB–X bonds, while a predominant electrostatic contribution (Δ*E*_elstat_) suggests an interaction that is more ionic in nature. Notably, the extent of covalency varies depending on the identity of the coordinated small molecule. Previous work by Braunschweig *et al.*,^61^ demonstrated that the boron centre in a certain borylene species could engage in metal-like π-back donation to ligands-behaviour traditionally associated with transition metals. The present EDA study reveals the similar behaviour from the borylene to various ligand systems across the complex 1–9, with variable degrees of orbital interaction, underscoring the bonding characteristics of these CAAC-stabilized borylene complexes.

**Table 2:** Energy decomposition analysis of complex 1–9. The calculations were carried out at BP86/TZ2P level using M06-2X/6-311G** optimized geometries. [M]–X where [M = NCB = CAAC–B–N(SiMe_3_)_2_] and X = C_2_H_4_ (1), CN^−^ (2), CO (3), N_2_ (4), NO^+^ (5). PH_3_ (6), C_6_H_6_ (7), CH_4_ (8) and H_2_ (9) (energy contributions given in kcal mol^−1^)

	[M]–CO	[M]–CN^−^	[M]–PH_3_	[M]–N_2_	[M]–C_2_H_4_	[M]–NO^+^	[M](H)–CH_3_	[M](H)–C_6_H_5_	[M](H)–(H)
Δ*E*_int_	−55.08	−123.78	−26.25	−31.25	−22.00	−135.60	−22.02	−134.68	−170.39
Δ*E*_Pauli_	246.63	152.56	169.47	217.92	537.60	560.84	323.24	601.29	484.44
Δ*E*_elect_^a^	−120.63 (39.98%)	−54.79 (19.83%)	−94.00 (47.17%)	−95.95 (38.51%)	−257.77 (46.06%)	−132.10 (18.97%)	−137.24 (39.75%)	−256.25 (34.82%)	−146.65 (22.40%)
Δ*E*_orb_^a^	−181.08 (60.02%)	−221.55 (80.17%)	−105.25 (52.83%)	−153.22 (61.49%)	−301.83 (53.94%)	−564.34 (81.03%)	−208.02 (60.25%)	−479.72 (65.18%)	−508.18 (77.60%)

a Values in parenthesis give percentage contributions to the total attractive interactions Δ*E*_elstst_ + Δ*E*_orb_.

The bonding interactions in NCB–X, which is given by Δ*E*_int_ increases for X with the order in type A complex follows the trends NCB–C_2_H_4_ < NCB–PH_3_ < NCB–N_2_ < NCB–CO < NCB–CN^−^ < NCB–NO^+^ and in type B complex follows NCB(H)–CH_3_ < NCB(H)–C_6_H_5_ < NCB(H)–(H). However, the trend of the Δ*E*_int_ value does not fully agree with the trend of the orbital interactions Δ*E*_orb_. The orbital term for NCB(H)–CH_3_ for example is larger (−208.02 kcal mol^−1^) than B–PH_3_ (−105.25 kcal mol^−1^) and B–CO (−181.08 kcal mol^−1^) although the overall attraction for NCB–CO is weaker than that of NCB(H)–CH_3_. It shows that the orbital interactions may not always be of precise probe for the trend of bonding interactions. Other factors such as the electrostatic attraction and Pauli repulsion may change the order of total interactions of a chemical bond. As the changes in covalent bonding are often, explained only in terms of orbital interactions.

**Fig. 8:**
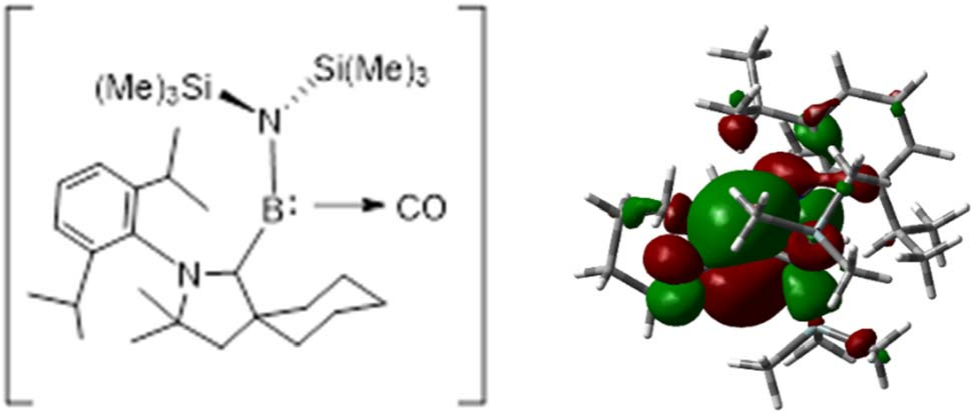
(Left) Schematic representation of the donor–acceptor bonds in NCB–CO. (Right) Plot of the HOMO of NCB–CO.

The bonding situation in the NCB–CO complex shows that, it has an interaction energy −55.08 kcal mol^−1^, and the orbital interactions contribute 60.02% of the attractive interactions, showing that covalent bonding dominates, while electrostatic interactions account for 39.98%, providing additional long-range stabilization. The Pauli repulsion of 246.63 kcal mol^−1^, is quite significant which arises from the overlapping of the occupied orbital between the borylene fragment with rich electron density and the CO adduct. Despite this significant repulsion, the strong orbital interactions overcome the destabilizing effect, resulting in a net stabilizing interaction. The bonding system in NCB–CO is determined by a balance between covalent orbital contributions, electrostatic stabilization, and short-range electronic repulsion. The activation of CO_2_ by using the frustrated Lewis pairs reported that the large frozen interaction energy reflects strong Pauli repulsion.^62^ Similarly, in the (BH)(CO)_2_ system,^63^ orbital interactions are the primary source of strong bonding characteristics which is largely driven by the significant CO → (BH) π-back donation (Fig. 8).

The NCB–N_2_ complex exhibits a moderate interaction energy of −31.25 kcal mol^−1^, with mostly the orbital interactions contributing 61.49% and electrostatic interactions of 38.51%. The Pauli repulsion is comparatively large with 217.92 kcal mol^−1^, reflecting short-range electron–electron repulsion between the filled orbitals of the electron-rich borylene centre and the π system N_2_ fragments. Although the interaction is net stabilizing, the relatively large Pauli term limits the overall binding strength, resulting in a weaker interaction compared to isoelectronic analogues.

In contrast, the NCB–CN^−^ complex displays a much stronger interaction of −123.78 kcal mol^−1^, dominated by orbital interactions with 80.17% and a smaller electrostatic contribution with 19.83%. The Pauli repulsion is comparatively lower with 152.56 kcal mol^−1^, this indicates the highly covalent nature of the NCB–CN^−^ bond, where the filled p-orbital on boron centre engages in strong charge donation into the π* orbital of CN^−^, leading to significant electron delocalization.

In the N_2_ complex, the weaker orbital interaction reflects the lower π* acceptor character of N_2_ compared to CN^−^, resulting in limited delocalization and only modest weakening of the N–N triple bond. These observations illustrate that the strength of NCB–ligand bonding is determined by a balance between orbital stabilization and Pauli repulsion.

These results are consistent with the observed high spin density localized at the terminal nitrogen of the N_2_ fragment, reflecting limited delocalization of electron density along the N–N triple bond.^64^ The weakening of N–N bond is promoted by a “pull” effect, leading to a reduction in its bond order.^65^

The bonding picture in the NCB–NO^+^ complex exhibits a strong interaction energy of −135.60 kcal mol^−1^, indicating highly stabilizing bonding between the borylene and the adduct. Orbital interactions dominate the attractive component, contributing 81.03% of the total stabilization, while electrostatic interactions account for only 18.97%, reflecting a largely covalent character of the NCB–NO^+^ bond. Despite this significant repulsion of 560.84 kcal mol^−1^, the strong orbital interactions, likely involving donation from the filled orbitals of boron into the vacant orbitals of NO^+^ and complementary back-donation from NO^+^ to the NCB fragment, overcome the destabilizing effect, resulting in a net strongly stabilizing interaction. The bonding can be described as highly covalent in nature, with short range repulsion balanced by extensive orbital mixing.

In the case of the NCB(H)–CH_3_ complex, the orbital interaction term contributes 60.25% of the total attractive interaction, exceeding the electrostatic contribution 39.75%. This balance indicates that the interaction is predominantly covalent in nature, with stabilization arising mainly from effective orbital overlap rather than long range electrostatics. The interaction is accompanied by a substantial Pauli repulsion term with 323.24 kcal mol^−1^, which originates from significant occupied–occupied orbital overlap between the electron-rich NCB(H) fragment and the approaching CH_3_ moiety. This large repulsive contribution reflects the close-contact geometry and electronic congestion inherent to C–H bond cleavage and B–C bond formation at the boron centre. However, the magnitude of the stabilizing orbital interactions is sufficient to overcome this Pauli repulsion energy, resulting in a net favourable interaction energy.

**Fig. 9:**
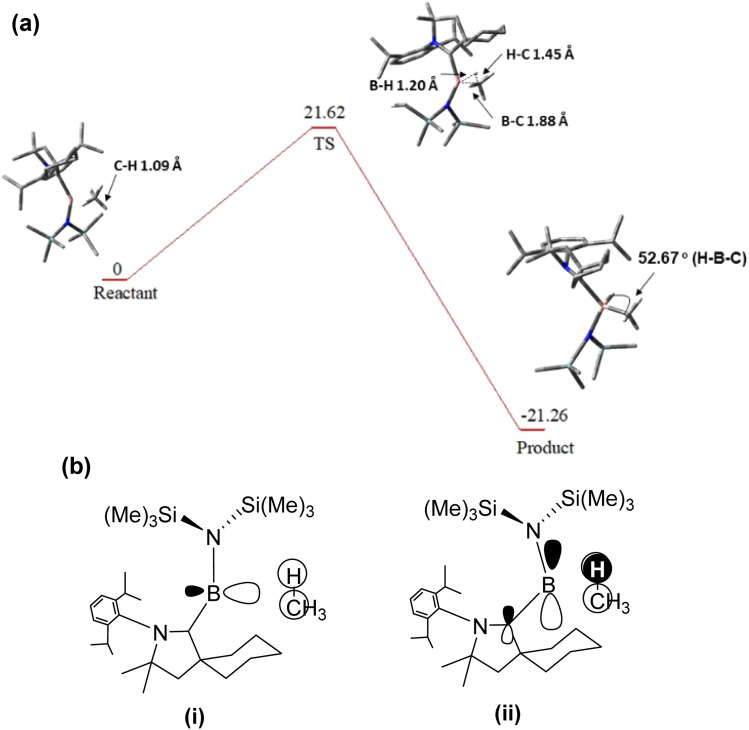
(a) Energy profile showing the TS for the activation of CH_4_. (b(i)) Primary interaction between the LUMO of bent form of NCB(H)–CH_3_ and the σ orbital of CH_4_ (ii) secondary interaction between the HOMO of the bent form of NCB(H)–CH_3_ and the σ* orbital of CH_4_. Hydrogen atoms are omitted for clarity.

The kinetic studies were conducted to evaluate the energetic involved during the transition state formation and the subsequent C–H bond cleavage in CH_4_. Fig. 9a presents the corresponding free energy profile, which reveals that the reaction proceeds through an endergonic transition state with a calculated free energy barrier of +21.62 kcal mol^−1^. This barrier reflects the substantial energy required to activate and cleave the strong C–H bond of methane. Following the transition state, the reaction becomes thermodynamically favourable, as the subsequent product formation is characterized by proton abstraction by the borylene centre and concomitant formation of a new B–H bond is highly exergonic, with a free energy change of +21.62 kcal mol^−1^. The significant stabilization associated with B–H bond formation effectively compensates for the high activation barrier, indicating that although the C–H activation step is kinetically demanding, the overall process is strongly thermodynamically favourable. Fig. 9b illustrates the bent arrangement in NCB(H)–CH_3_ complex which facilitates efficient donor–acceptor interactions while minimizing Pauli repulsion, highlighting how strong covalent orbital interactions can dominate the bonding even in the presence of significant steric and electronic repulsion.

In contrast, the NCB–C_2_H_4_ complex exhibits a relatively larger electrostatic contribution of 46.06% and a smaller orbital interaction component with 53.94% together with a modest overall interaction energy of −22.00 kcal mol^−1^. This balance indicates that the interaction is largely governed by electrostatic attraction, with limited covalent character. Notably, the interaction is associated with a very large Pauli repulsion term 537.60 kcal mol^−1^, which originates from orbital overlap between the filled π-system of ethylene and the borylene fragment. This substantial short-range repulsion strongly counteracts the stabilizing electrostatic and orbital contributions. As a result, the activation of the ethylene π-system remains weak, and the bonding interaction is significantly less substantial than in the CH_3_ or H_2_ complexes. These results illustrate that, in systems with extended π-electron density such as ethylene, large Pauli repulsion can effectively suppress orbital stabilization, leading to modest binding even when electrostatic contributions are non-negligible.

The bonding in the NCB(H)–C_6_H_5_ complex is dominated by orbital interactions (65.18%), with a smaller electrostatic contribution 34.82%, yielding a substantial overall interaction energy of −134.68 kcal mol^−1^. Despite this net stabilization, the complex exhibits the largest Pauli repulsion among the studied systems with 601.29 kcal mol^−1^, originating from significant occupied orbital overlap between the electron-rich borylene fragment and the π-electrons of benzene. This high repulsive energy reflects both steric congestion and electron–electron repulsion within the planar aromatic framework, highlighting that close approach of the fragments introduces strong short range electronic destabilization. Nevertheless, the dominant orbital interactions effectively overcome this Pauli repulsion resulting in a strongly stabilizing net interaction.

**Fig. 10:**
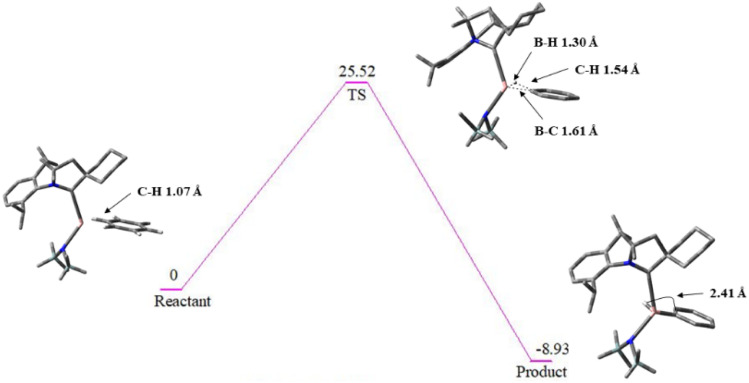
Energy profile showing the TS for the activation of benzene, cleavage of C–H bond and the formation of new B–H bond.

The balance between the substantial repulsive and stabilizing components suggests that the borylene fragment perturbs the π-system of benzene without complete disruption of aromaticity, forming a π-coordinated adduct reminiscent to that of transition metal complexes.^66,67^ Overall, the EDA highlights how extremely large Pauli repulsion does not preclude strong bonding when compensated by favourable orbital interactions, emphasizing the critical role of orbital contributions in governing both the stability and potential reactivity of the NCB(H)–C_6_H_5_ complex. Fig. 10 presents the reaction pathway for the activation of C_6_H_6_ which proceeds *via* a well-defined transition state involving C–H bond cleavage and the formation of a new B–H bond with a bond length of 1.30 A. The transition state is endergonic, with a calculated free energy barrier of +25.52 kcal mol^−1^ reflecting the energetic cost of aromatic C–H bond activation. The inclusion of zero-point corrections and the thermal corrections slightly reduce the barrier height but do not alter the overall trend. The subsequent product formation is exergonic by −8.93 kcal mol^−1^, indicating that despite the kinetically demanding nature of the process, though the transformation is thermodynamically feasible.

The energy decomposition analysis of the NCB(H)–(H) complex reveals a strongly stabilizing interaction energy −170.39 kcal mol^−1^, despite the presence of a very large Pauli repulsion 484.44 kcal mol^−1^. This substantial Pauli repulsion originates from significant occupied–occupied orbital overlap during the close approach of the H_2_ fragment to the electron rich borylene centre, reflecting an electronic congestion in the interaction region.

**Fig. 11:**
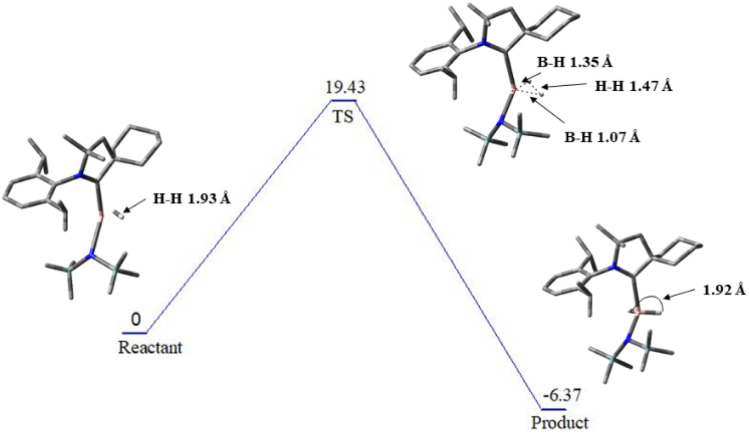
Energy profile showing the TS for the activation of H_2_.

This destabilizing contribution is more than compensated by a dominant orbital interaction term, which accounts for 77.60% of the total attractive interactions, while electrostatic interactions contribute 22.40%. The overwhelming magnitude of Δ*E*_orb_ highlights the strong covalent character of the NCB(H)–(H) interaction, arising from efficient electron-sharing and favourable opposite-spin electron pairing during H_2_ activation. As a result, the net interaction energy remains highly stabilizing despite the large Pauli penalty. Fig. 11 illustrates the energy profile associated with H–H bond cleavage. The transition state is endergonic, with a free energy barrier of +19.43 kcal mol^−1^, while the subsequent formation of two B–H bonds is thermodynamically favourable, exhibiting an exergonic free energy change of −6.37 kcal mol^−1^.

This balance between strong Pauli repulsion and even stronger orbital stabilization illustrates that trends in Δ*E*_int_ cannot be inferred from individual EDA components in isolation. Similar behaviour has been reported by Zhao *et al.*^54^ for B–H_2_ systems, where large Pauli repulsion accompanying close-contact geometries is offset by substantial orbital interactions.

Furthermore, the facile H_2_ splitting *via* nucleophilic activation at a single carbene carbon reported by Guido *et al.*^68^ is consistent with this bonding picture unlike electrophilic transition metals, nucleophilic carbenes promote hydride-like character in one hydrogen atom, enabling strong orbital interactions that dominate the overall stabilization.

The NCB–PH_3_ complex demonstrates bonding characteristics akin to those in Cl_3_B–PH_3_, where Celik *et al.*, found greater orbital contributions but weaker electrostatic interactions due to phosphorus having only a half-filled p orbital.^69^ The EDA value shows that the total interaction energy of B–P bond is Δ*E*_int_ = −26.25 kcal mol^−1^ comparatively smaller than that of B–P bond from Cl_3_B–PH_3_ (−41.3 kcal mol^−1^), which comprises attractive electrostatic of −94.00 kcal mol^−1^ (47.17%) and orbital interactions of −105.25 kcal mol^−1^ (52.83%) and a large Pauli repulsion of 169.47 kcal mol^−1^. The latter term even overcompensates the orbital stabilization without the attractive contributions arising from quasiclassical electrostatic interactions. The Pauli repulsion term measures steric and electron–electron repulsion between filled orbitals and plays a key role in modulating Δ*E*_int_. Large Pauli contributions are observed for sterically congested complexes, such as NCB(H)–C_6_H_5_ (601.29 kcal mol^−1^) and NCB–C_2_H_4_ (537.60 kcal mol^−1^), where filled orbitals of the ligand and boron centre interact unfavourably. Similarly, in NCB(H)–H_2_ the Pauli repulsions term (484.44 kcal mol^−1^) reflects close approach of the H_2_ σ orbital to filled boron orbitals. In contrast, NCB–CN^−^ and NCB–PH_3_ exhibit lower Pauli repulsion (152.56 and 169.47), consistent with better electronic complementarity. These observations demonstrate that Pauli repulsion can reduce overall bonding strength and influence the geometry and accessibility of the boron centre for the ligand activation.

The EDA shows that bonding in borylene complexes arises from a delicate balance between covalent orbital interactions, electrostatics, and Pauli repulsion. Strong donor–acceptor complexes exhibit high orbital contributions and significant back-donation, while weaker donors rely more on electrostatics, and Pauli repulsion modulates the overall interaction strength, particularly in sterically congested systems. This systematic analysis provides a clear chemical rationale for the observed trends in stability and reactivity across the series.

## 4. Conclusion

In summary, density functional theory (DFT) calculations employing the M06-2X functional provided a valuable insight into the bonding and activation processes of small molecules by the CAAC-stabilized borylene (NCB). These computational studies underscore the pivotal role of donor–acceptor interactions in the activation of small molecules by main-group species, which is reminiscent of the transition metal-mediated activation of small molecules. In both the type A and type B complexes, the bonding in the NCB–X adducts can be effectively described in terms of σ-donation (X → NCB) and π-back donation (X ← NCB), where the σ-donation enhances the overall binding affinity, while the π-back donation plays a critical role in weakening and thereby activating the coordinated small molecule. This donor–acceptor framework provides a deeper understanding of the structure–reactivity relationships in borylene-mediated small molecule activation and offers a guiding principle for the design of next-generation main-group-based activating agents, offering a compelling alternative to transition metals.

## Author contributions

Vikiho Wotsa: data curation; formal analysis; investigation. Chinnappan Sivasankar: conceptualization; formal analysis; funding acquisition; methodology; project administration; supervision.

## Conflicts of interest

There is no conflict of interest to declare.

## Supplementary Material

RA-016-D6RA00186F-s001

## Data Availability

The data that support the findings of this study are available in the supplementary information (SI) of this article. Supplementary information: , calculated energies, bond parameters, molecular geometries and Cartesian coordinates. See DOI: https://doi.org/10.1039/d6ra00186f.
