# Supplementary material for: Electronic structure analysis of borylenes and their role in small molecule activation
Source: RSC Adv. 2026 Feb 20;16(11):10284–96. doi: 10.1039/d6ra00186f (PMC12922940; doi:10.1039/d6ra00186f)
Supplement: RA-016-D6RA00186F-s001 [file RA-016-D6RA00186F-s001.pdf]

Supplementary Information (SI) for RSC Advances.
This journal is © The Royal Society of Chemistry 2026

Electronic Structure Analysis of Borylenes and Their Role in Small Molecule Activation

Supporting information:

Table S1. Calculated energies and entropies for the complexes **1-9.**

Sl. No. Systems	Symmetry point Group	Energy [Hartree]	Zero point correction [Hartree]	Entropy [kcal mol^{-1}]	*G* [kcal mol^{-1}]
1. **NCB-C_2H_4 (1)**	C_1	- 1926.401593	0.833282	0.875791	-1208817.83
**2. NCB-CN^- (2)**	C_1	-1941.079579	0.783308	0.825416	-1218027.44
**3. NCB-CO (3)**	C_1	- 1961.476442	0.785690	0.828241	-1230826.47
4. **NCB-N_2 (4)**	C_1	-1957.646116	0.786898	0.828241	-1228422.94
**5. NCB-NO^+ (5)**	C_1	-1977.870614	0.787474	0.720103	-1241113.81
**6. NCB-PH_3 (6)**	C_1	-2191.234439	0.803992	0.846633	-1374999.61
**7. NCB(H)-C_6H_5 (7)**	C_1	-2080.103031	0.878828	0.923638	-1305264.91
**8. NCB(H)-CH_3 (8)**	C_1	-1887.914973	0.831627	0.873903	-1184666.65
**9. NCB(H)-(H) (9)**	C_1	-1849.330176	0.796209	0.836466	-1160454.69

Table S2. Calculated bond lengths (B-L1 and B-L2) (Å) and bond angles (°) of complexes **1-9** [CAAC-B-N($SiMe_3$)$_2$] where [L1 = CAAC and L2 = N($SiMe_3$)$_2$].

System	Bond length (Å)		Bond angle (°)
	B-L1	B-L2	C-B-N
**NCB*** (calc.)	1.40 [1.41] [a]	1.38 [1.38] [a]	175.01 [175.4] [a]
**NCB*** (exp.)	[1.401(5)]	[1.382(5)]	[174.8(3)]
**NCB-C_2H_4 (1)**	1.63	1.43	123.49
**NCB-CN^- (2)**	1.46	1.54	130.85
**NCB-CO (3)**	1.51	1.50	134.73
**NCB-CO***	1.510(5)	1.530(5)	129.10(3)
**NCB-N_2 (4)**	1.51	1.50	137.01
**NCB-NO^+ (5)**	1.60	1.44	155.39
**NCB-PH_3 (6)**	1.46	1.50	134.73
**NCB(H)-C_6H_5 (7)**	1.57	1.53	129.54
**NCB(H)-CH_3 (8)**	1.58	1.43	125.39
**NCB-(H)(H) (9)**	1.62	1.55	119.58

* Borylene compound calculated and experimental values

[a] B3LYP/G-311G** calculated bond lengths and bond angle.

Table S3. Selected natural population analysis charges of complex **1-9**.

**Entry**	$_{31}$**B**	$_{4}$**C**	$_{88}$**C**	$_{61}$**N**	$_{88}$**N**	$_{89}$**N**	$_{89}$**O**	$_{88}$**P**	$_{88}$**H**	$_{89}$**H**	$_{91}$**C**
**NCB-C_2H_4**	-1.036	0.171	0.237	1.571	-	-	-	-	-	-	0.853
**NCB-CN^-**	-0.383	0.246	-0.091	1.560	0.480	-	-	-	-	-	-
**NCB-CO**	-0.173	-0.063	-0.573	1.519	-	-	0.452	-	-	-	-
**NCB-N_2**	-0.489	0.014	-	1.523	0.074	-0.020	-	-	-	-	-
**NCB-NO^+**	-0.763	-0.216	-	1.481	-0.021	-	2.186	-	-	-	-
**NCB-PH_3**	-0.268	0.209	-	1.574	-	-	-	-0.467	-	-	-
**NCB(H)-C_6H_5**	-0.484	-0.385	0.296	1.563	-	-	-	-	-0.123	-	-
**NCB(H)-CH_3**	-0.520	-0.165	0.987	1.526	-	-	-	-	-0.208	-	-
**NCB(H)-(H)**	-0.005	-0.345	-	1.558	-	-	-	-	0.005	0.049	-

Table S4. Calculated NBO and WBI of complex **1-9** [**NCB** = CAAC-B-N($SiMe_3$)$_2$].

**System**	**Bond order**		**Occupancy**		**Wiberg Bond Indices**	
**NCB-C_2H_4**	1 (B-C)	1 (C_α-C_β)	0.97 (B-C)	0.98 (C_α-C_β)	0.82 (B-C)	1.07 (C_α-C_β)
**NCB(H)-C_6H_5**	1 (B-C)	1 (B-H)	1.95 (B-C)	1.53 (B-H)	0.84 (B-C)	0.56 (B-H)
**NCB(H)-CH_3**	1 (B-C)	1 (B-H)	0.98 (B-C)	0.94 (B-H)	0.49 (B-C)	0.52 (B-H)
**NCB-CN**	1 (B-C)	3 (C-N)	1.97 (B-C)	1.99 (C-N)	0.94 (B-C)	2.82 (C-N)
**NCB-CO**	1 (B-C)	2 (C-O)	1.94 (B-C)	1.99 (C-O)	1.14 (B-C)	-0.01 (C-O)
**NCB(H)-(H)**	1 (B-H_α)	1 (B-H_β)	1.92 (B-H_α)	1.96 (B-H_β)	0.90 (B-H_α)	0.93 (B-H_β)
**NCB-N_2**	1 (B-N)	2 (N_α-N_β)	1.98 (B-N)	1.99 (N_α-N_β)	0.90(B-N)	2.51 (N_α-N_β)
**NCB-NO^+**	1 (B-N)	1 (N-O)	1.97 (B-N)	1.99 (N-O)	1.16 (B-N)	1.92 (N-O)
**NCB-PH_3**	1 (B-P)	-	1.83 (B-P)	-	0.90 (B-P)	-

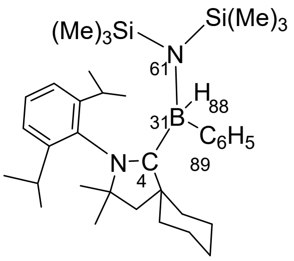

CBN-C_6H_6

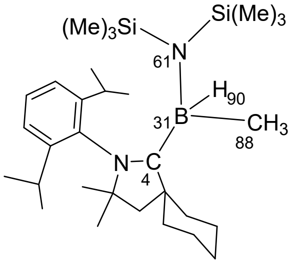

CBN-CH_4

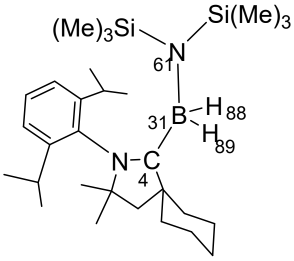

CBN-H_2

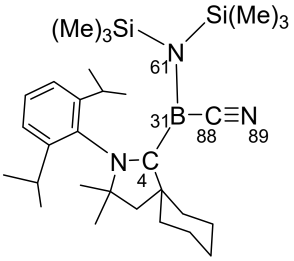

CBN-CN^-

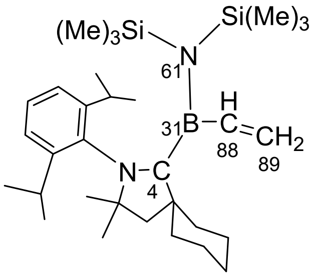

CBN-C_2H_4

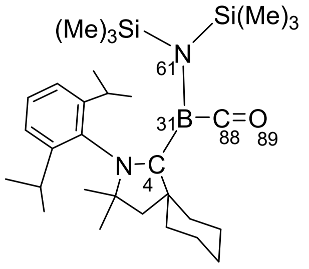

CBN-CO

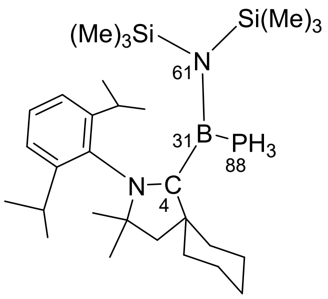

CBN-PH_3

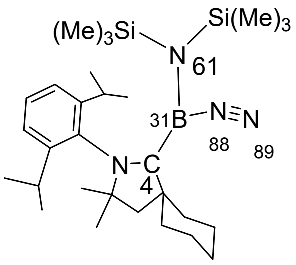

CBN-N_2

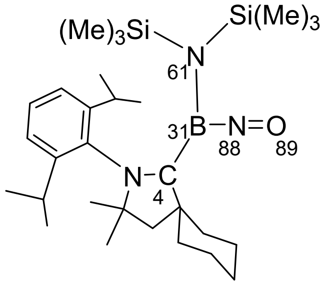

CBN-NO^+

Figure S1. Geometrical structures of complex **1-9**.

Figure S2. Cartesian coordinates of the optimized geometries of complex **1-9**. (Hydrogen atoms are removed for clarity except in complex **8** and **9**)

NCB-C_2H_4 (1)

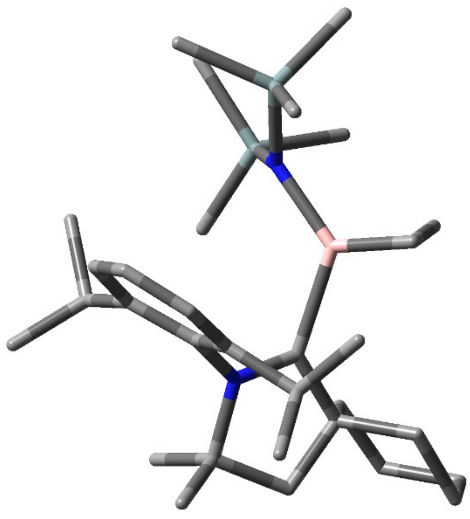

C	2.06312566	1.28428828	1.62221328
C	3.19998166	0.48652828	0.99591028
C	2.56059466	-0.64733572	0.16394428
C	1.10196666	-0.18454172	0.00816628
N	0.91300366	0.92381028	0.67743128
H	3.78457666	1.14049228	0.34248328
C	2.65541766	-2.04021272	0.83123228
C	4.09031466	-2.56744872	0.92187928
C	4.80618966	-2.54632072	-0.42706372
C	4.75294466	-1.14673072	-1.03782972
C	3.30189666	-0.68719372	-1.20426972
H	5.84322866	-2.87131772	-0.30837872
H	4.66131366	-1.96626272	1.64002328

H	4.06197666	-3.58222172	1.32868528
H	2.04614666	-2.74348972	0.25225128
H	2.22545066	-2.00964572	1.83403428
H	5.31299066	-0.44619972	-0.40699572
H	5.23638366	-1.13492672	-2.01881772
H	3.26443966	0.30376428	-1.67012872
H	2.83294766	-1.38195872	-1.89583172
H	4.32798766	-3.26035372	-1.11097472
H	3.88071866	0.10884628	1.75918228
C	-1.27546634	1.96959228	1.29752128
C	-0.17720134	1.86859228	0.42529828
C	-0.02943434	2.73723828	-0.67962272
C	-1.03434434	3.67772228	-0.90597572
C	-2.14281834	3.76755628	-0.08335172
C	-2.25364334	2.91905628	1.00389328
H	-0.94353334	4.35321328	-1.74933972
H	-2.91271434	4.50448328	-0.28302472
H	-3.11166334	3.00908728	1.66083728
C	1.15665666	2.75954228	-1.63569072
C	-1.43130934	1.18179828	2.58482928
H	1.87785566	1.99695428	-1.32808072
H	-0.63949934	0.43577128	2.62703128
C	0.72325766	2.44978128	-3.07659372
H	0.17963066	3.29481128	-3.50821472
H	1.59856366	2.26835828	-3.70832472
H	0.06656266	1.58564728	-3.13161072
C	1.87447466	4.12197028	-1.62797672
H	2.83181866	4.04563128	-2.15173472
H	1.27740666	4.87525528	-2.14965572
H	2.05930066	4.50141628	-0.62193972
C	-1.30044634	2.12582228	3.79260428

H	-2.18255234	2.76779028	3.87568328
H	-1.22000434	1.55144928	4.72046528
H	-0.43195934	2.78663828	3.71827228
C	-2.75761834	0.42576228	2.66881128
H	-3.61670834	1.10275928	2.65479028
H	-2.85934134	-0.26762872	1.83742028
H	-2.80690834	-0.13842472	3.60529128
C	2.32920366	2.78057528	1.64917728
H	1.45108966	3.34498828	1.97281028
H	3.13510666	2.97296328	2.36192228
H	2.65208366	3.14929228	0.67713428
C	1.79477266	0.79487228	3.04981028
H	2.72616866	0.90503428	3.61117128
H	1.03909966	1.38591328	3.55995328
H	1.50827366	-0.25679572	3.08882928
B	-0.08110534	-0.86679072	-0.92025772
N	-1.39061934	-1.21179672	-0.36934772
Si	-1.46675634	-2.58034272	0.77024028
Si	-2.86128434	-0.82772872	-1.32079872
C	-0.54087734	-2.23421672	2.37041128
H	0.34039466	-1.61266472	2.22456928
H	-1.18358134	-1.73649772	3.09982128
H	-0.20757834	-3.17894072	2.81229028
C	-0.73558534	-4.15553172	0.02849528
H	-1.28323534	-4.47626872	-0.86454272
H	0.32490266	-4.09723772	-0.23236072
H	-0.83603434	-4.95738072	0.76827728
C	-3.21121934	-3.06660372	1.26600028
H	-3.86715734	-3.31246972	0.42697428
H	-3.10399434	-3.97943772	1.86399228
H	-3.71282634	-2.32912172	1.89547128

C	-4.37205334	-0.38364572	-0.29498472
H	-4.72620334	-1.13174772	0.41240428
H	-4.20724034	0.55431128	0.24227728
H	-5.18567034	-0.20285072	-1.00768672
C	-3.26894734	-2.26949072	-2.46037772
H	-2.53489734	-2.37332772	-3.26769172
H	-3.30451534	-3.22301072	-1.92309872
H	-4.24724634	-2.12371872	-2.93028072
C	-2.67212534	0.75391628	-2.33049572
H	-2.48119134	1.59882528	-1.66296272
H	-1.94451634	0.80023628	-3.14026772
H	-3.64917734	0.93085728	-2.79429772
X	-0.07619134	-1.56854715	-2.26855805
C	-0.00050049	-2.14188390	-1.75423602
H	0.88804698	-2.26611319	-1.30484701
H	-0.76490060	-2.74538873	-1.52735641
C	-0.16688687	-1.04592693	-2.76477745
H	-1.02799844	-0.86942032	-3.24875362
H	0.65086854	-0.49365884	-2.92687612

NCB-CN^{-} (**2**)

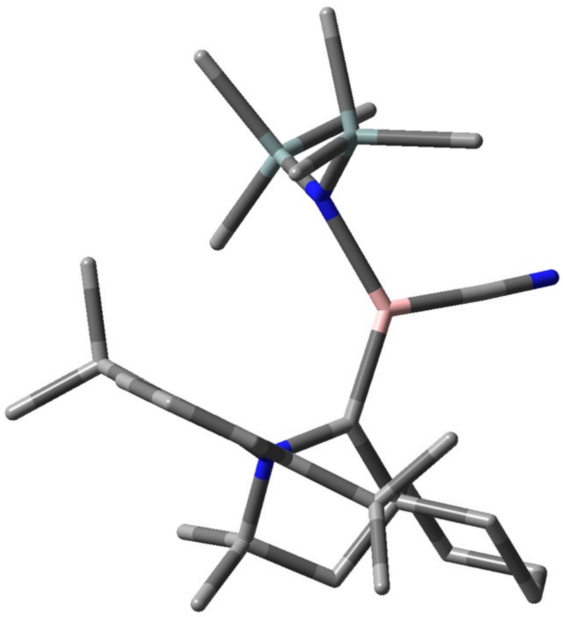

C	-1.85317600	1.39505900	-1.52101800
C	-3.04156200	0.64754200	-0.89107300
C	-2.48125200	-0.63569200	-0.23820300
C	-0.96635200	-0.32971200	-0.08032200
N	-0.71145000	0.95923200	-0.68685500
H	-3.49557400	1.28751300	-0.12587200
C	-2.68850700	-1.86559500	-1.16387900
C	-4.15140800	-2.27027600	-1.36366000
C	-4.84469100	-2.50914200	-0.02323800
C	-4.70304900	-1.28265600	0.87703800
C	-3.22934300	-0.92145500	1.08785400
H	-5.90166600	-2.75874500	-0.17415200
H	-4.69477300	-1.49188700	-1.91607100
H	-4.19711800	-3.17457200	-1.98116600
H	-2.15453600	-2.70836900	-0.71886800
H	-2.21548800	-1.68126400	-2.13111900
H	-5.23812900	-0.43626700	0.42403800
H	-5.17759800	-1.46853500	1.84727100
H	-3.15841500	-0.05483900	1.75065600
H	-2.73903200	-1.74911900	1.60293800
H	-4.37461100	-3.36757300	0.47238000
H	-3.82053200	0.44619900	-1.63135600
C	-2.09389700	2.90564100	-1.48933200
H	-1.24813300	3.46035100	-1.90340900
H	-2.98052800	3.13601100	-2.08810700
H	-2.26524600	3.26636200	-0.47404100
C	-1.66699700	0.97800800	-2.99169400
H	-2.58415500	1.17066200	-3.55957900

H	-0.85588600	1.54118900	-3.45887000
H	-1.43124000	-0.08216000	-3.07638200
B	0.02978900	-1.27488300	0.41038400
C	1.96763000	3.49953600	-0.22152100
C	1.21686200	2.45834500	-0.76665200
C	0.10607100	1.94800900	-0.06596900
C	-0.21001300	2.50575100	1.19627400
C	0.57732500	3.53033900	1.71284500
C	1.65867300	4.03903400	1.01202900
H	2.82083500	3.88200400	-0.77395900
H	0.33132300	3.94663700	2.68489300
H	2.25620200	4.84291500	1.42917700
C	-1.39827300	2.03620400	2.01003900
C	1.64755300	1.88593300	-2.10058300
H	-1.98927800	1.40141400	1.35991000
H	0.96163100	1.07176000	-2.32143700
C	3.05162100	1.28811900	-2.00157700
H	3.08918800	0.58856100	-1.16614000
H	3.30686000	0.74638500	-2.91819800
H	3.81089400	2.06202500	-1.84148100
C	1.56238300	2.91272600	-3.23364500
H	1.79786800	2.44254300	-4.19425600
H	0.56248200	3.34936800	-3.30621800
H	2.27190300	3.73334500	-3.08063000
C	-2.30110500	3.18419600	2.47462700
H	-2.59079600	3.83387500	1.64389200
H	-3.21353700	2.77821700	2.92339200
H	-1.81397400	3.80804400	3.23091700
C	-0.95522000	1.17615600	3.19572300
H	-0.41566200	0.30082400	2.83285500
H	-0.30540200	1.74253000	3.87206700

H	-1.82330000	0.82630800	3.76429800
N	1.55977400	-1.30139300	0.24787500
Si	1.98159000	-2.28912900	-1.12435200
Si	2.51888000	-1.06803800	1.67619600
C	3.81148800	-2.22035200	-1.62569500
H	4.43114000	-2.86290400	-0.99647600
H	3.88210800	-2.60239600	-2.65091300
H	4.24376600	-1.21818600	-1.61710800
C	1.67111400	-4.12743400	-0.82594100
H	1.98378200	-4.71124800	-1.70017300
H	2.24066500	-4.48084900	0.04018000
H	0.61788100	-4.33393100	-0.62413000
C	0.97931300	-1.75894600	-2.63297100
H	0.45377600	-0.83497100	-2.39703000
H	1.61498900	-1.59565500	-3.50974400
H	0.22286400	-2.50673600	-2.88839100
C	4.23308300	-1.86322200	1.56080400
H	4.18074500	-2.93784200	1.36142900
H	4.86851100	-1.40344600	0.80025500
H	4.72613200	-1.73110800	2.53062700
C	1.74404300	-1.83265000	3.21539600
H	1.61086200	-2.91056700	3.09080900
H	2.39591100	-1.65379300	4.07843900
H	0.75872000	-1.42021100	3.44201300
C	2.82908000	0.74768800	2.04070000
H	3.32189000	1.24679900	1.20182400
H	1.89558000	1.28024300	2.22673700
H	3.46496900	0.85466800	2.92720300
C	-0.52198200	-2.56987900	1.11725300
N	-0.88987500	-3.54390500	1.62865000

NCB-CO (**3**)

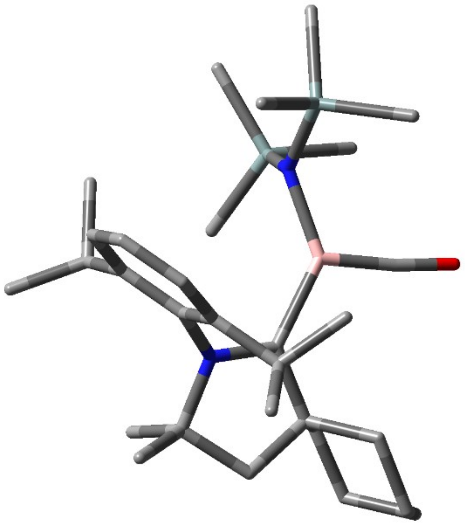

C	2.03756000	1.40243300	1.41638900
C	3.15370500	0.54457500	0.81759600
C	2.48350600	-0.70731100	0.21008400
C	1.00375200	-0.27763700	0.06163200
N	0.86269000	0.97491300	0.59662000
H	3.66060500	1.11220200	0.03126600
C	2.61766700	-1.94209900	1.13737200
C	4.05663600	-2.43677800	1.29378400
C	4.70835000	-2.70140000	-0.06293600
C	4.61582700	-1.47020300	-0.96331500
C	3.16019900	-1.03246000	-1.14497100
H	5.75217100	-3.00102900	0.06882500
H	4.65550700	-1.70341300	1.84667800
H	4.05691000	-3.34829500	1.89886500
H	2.01342900	-2.75266800	0.72042700
H	2.18388200	-1.72239400	2.11573100
H	5.20460700	-0.65293200	-0.52887000
H	5.05473800	-1.68116900	-1.94279700

H	3.10403000	-0.16070000	-1.80216000
H	2.63449800	-1.83802700	-1.65882400
H	4.19593000	-3.54002200	-0.55119900
H	3.90681200	0.29874000	1.56682900
C	2.33851000	2.89323000	1.29157600
H	1.51781200	3.50437900	1.67378500
H	3.23385000	3.11958600	1.87618400
H	2.52725000	3.18248000	0.25765300
C	1.82859100	1.06532000	2.90016700
H	2.76409300	1.22733600	3.44292000
H	1.06843600	1.70156500	3.35170900
H	1.53134900	0.02562700	3.04162300
B	-0.09314500	-1.15780900	-0.45702400
C	-1.92555200	3.45562300	0.46363500
C	-1.13567300	2.39797800	0.91402400
C	-0.06909600	1.96187700	0.11631300
C	0.19372100	2.58577500	-1.12353600
C	-0.62946700	3.62957200	-1.53428100
C	-1.68012700	4.07118500	-0.74791200
H	-2.75237500	3.79708300	1.07698400
H	-0.44362800	4.10938000	-2.48839500
H	-2.30715400	4.88937600	-1.08348100
C	1.33493300	2.16502300	-2.03434400
C	-1.47930900	1.76572800	2.24544100
H	2.01734700	1.55637300	-1.44450100
H	-0.77346200	0.95540100	2.41499000
C	-2.87917900	1.15056200	2.20006800
H	-2.97252300	0.50997900	1.32349200
H	-3.06531900	0.54929400	3.09457900
H	-3.65345800	1.92222500	2.14677700
C	-1.35915100	2.76558300	3.40104900

H	-1.49611800	2.25889500	4.36078900
H	-0.38527600	3.26196200	3.41167700
H	-2.12200300	3.54608100	3.32622300
C	2.14367600	3.34999100	-2.57426900
H	2.45094400	4.03282200	-1.77806000
H	3.04337700	2.98456700	-3.07725000
H	1.57630400	3.92937100	-3.30770300
C	0.82329800	1.29458400	-3.18701700
H	0.32325400	0.40519400	-2.80227600
H	0.11495500	1.85113300	-3.80866200
H	1.65305200	0.97250300	-3.82379800
N	-1.59384100	-1.22757900	-0.24962600
Si	-1.96480300	-2.30758900	1.09115000
Si	-2.63564900	-0.92412500	-1.62917500
C	-3.75575500	-2.26373000	1.68470300
H	-4.40899300	-2.87284900	1.05766500
H	-3.77215600	-2.70211000	2.68875600
H	-4.18922200	-1.26558600	1.75736200
C	-1.66667000	-4.11190600	0.62754100
H	-2.01417700	-4.76312800	1.43688000
H	-2.22119900	-4.38395200	-0.27663900
H	-0.61236000	-4.33950000	0.45241900
C	-0.86146700	-1.89104800	2.56664200
H	-0.30170000	-0.97454700	2.38514200
H	-1.45225900	-1.75415100	3.47708800
H	-0.13449000	-2.68641100	2.75340700
C	-4.33846500	-1.70137000	-1.43110100
H	-4.29434500	-2.78332700	-1.27793900
H	-4.91961100	-1.26307000	-0.61721500
H	-4.88870800	-1.52601100	-2.36166900
C	-1.92608700	-1.67327400	-3.20667700

H	-1.82304800	-2.75929700	-3.12192700
H	-2.60786600	-1.46813100	-4.03862200
H	-0.94822000	-1.26693600	-3.47596700
C	-2.89993900	0.90568200	-1.91299900
H	-3.31593900	1.38960100	-1.02565300
H	-1.96799700	1.41710900	-2.15572500
H	-3.59702500	1.06015400	-2.74335400
C	0.40422400	-2.36303000	-1.16916800
O	0.67453700	-3.31923900	-1.75105900

NCB-N_2 (**4**)

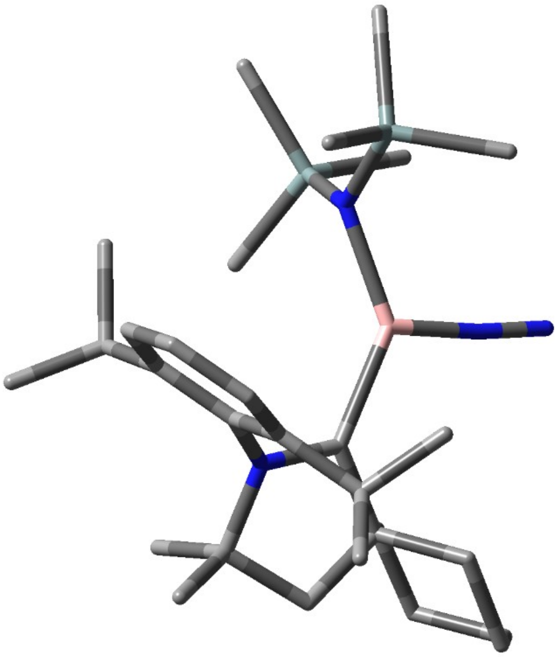

C	-1.98888600	1.34061100	-1.48083500
C	-3.12020300	0.48835500	-0.89947800
C	-2.46780400	-0.74062000	-0.22633700
C	-0.98739300	-0.31256200	-0.07022300
N	-0.83256800	0.94218700	-0.62688600
H	-3.67129300	1.07444100	-0.15795200
C	-2.58623000	-2.00489700	-1.11638700

C	-4.02075300	-2.51130500	-1.27826400
C	-4.69125100	-2.73390100	0.07598200
C	-4.62900500	-1.46571800	0.92582000
C	-3.18213400	-1.00601600	1.12283700
H	-5.72878800	-3.05248400	-0.06070300
H	-4.61414000	-1.79764600	-1.86190000
H	-4.00858600	-3.44159100	-1.85384000
H	-1.98723000	-2.80643100	-0.67693900
H	-2.14600400	-1.80662700	-2.09670700
H	-5.21478500	-0.67413600	0.44230000
H	-5.08878300	-1.63899100	1.90324700
H	-3.15523700	-0.10042300	1.73307500
H	-2.66394200	-1.77542500	1.69456600
H	-4.17594800	-3.54604300	0.60460100
H	-3.83557000	0.20842500	-1.67386100
C	-2.30074500	2.83266400	-1.39671500
H	-1.47317100	3.43813800	-1.77346900
H	-3.18272100	3.04321100	-2.00680100
H	-2.51266800	3.14466600	-0.37339000
C	-1.74529300	0.97144100	-2.95200300
H	-2.65374100	1.16155200	-3.53081000
H	-0.94243500	1.56377000	-3.38757900
H	-1.48784800	-0.08187600	-3.06608300
B	0.11456900	-1.16187600	0.44731100
C	1.90034300	3.47300500	-0.45875500
C	1.14567700	2.38625400	-0.89920200
C	0.05884300	1.95260500	-0.12487400
C	-0.24988700	2.60726900	1.08888100
C	0.54439700	3.67553000	1.49446200
C	1.60956700	4.11611200	0.72755900
H	2.73492600	3.81708500	-1.05992500

H	0.32173400	4.17761300	2.42894400
H	2.20991600	4.95623300	1.05771200
C	-1.41452900	2.19762200	1.97339800
C	1.53855100	1.72821400	-2.20512000
H	-2.09110000	1.60212800	1.36242100
H	0.87416800	0.87873200	-2.35400000
C	2.96753000	1.18798200	-2.13170200
H	3.06194000	0.50094500	-1.29169400
H	3.22526600	0.65454100	-3.05141000
H	3.69558400	1.99416000	-2.00052100
C	1.38781100	2.69067400	-3.38939300
H	1.52877900	2.16044700	-4.33589100
H	0.40378500	3.16590200	-3.40652600
H	2.13417100	3.48935300	-3.34147800
C	-2.22580600	3.38601400	2.49999300
H	-2.52296400	4.06467400	1.69625400
H	-3.13231500	3.02314600	2.99259800
H	-1.66829900	3.96761300	3.23915000
C	-0.93650100	1.31809600	3.13396900
H	-0.42334800	0.43369300	2.75550800
H	-0.24630700	1.87108400	3.77875500
H	-1.78265100	0.98873700	3.74463900
N	1.59901500	-1.23663600	0.28603600
Si	2.03099600	-2.28267300	-1.06427200
Si	2.57815300	-0.92107300	1.71152000
C	3.85386900	-2.27544500	-1.53950100
H	4.46454400	-2.88790500	-0.87419200
H	3.91892000	-2.71982100	-2.53927400
H	4.29996600	-1.28150000	-1.59684300
C	1.63034800	-4.08330100	-0.67145300
H	2.01714100	-4.73106100	-1.46513600

H	2.09516400	-4.39689000	0.26897200
H	0.55603500	-4.26944800	-0.59002200
C	1.05726200	-1.79371700	-2.60225300
H	0.18243900	-1.19996000	-2.33969400
H	1.67084800	-1.21279000	-3.29660400
H	0.70697200	-2.68673100	-3.12863300
C	4.28333300	-1.70703700	1.61153800
H	4.24010300	-2.78942800	1.46280400
H	4.91436200	-1.27725700	0.83109400
H	4.77988500	-1.53143400	2.57178600
C	1.78342800	-1.64678900	3.25904000
H	1.73440400	-2.73888500	3.21649500
H	2.39239100	-1.37700200	4.12817900
H	0.77312500	-1.27278400	3.44276400
C	2.81948500	0.91055000	1.99771500
H	3.21758900	1.40893500	1.11145000
H	1.88091200	1.40478900	2.25379900
H	3.52171500	1.07040000	2.82264500
N	-0.40874900	-2.35167000	1.13913200
N	-0.70798100	-3.26790300	1.70712900

NCB-NO$^+$ (**5**)

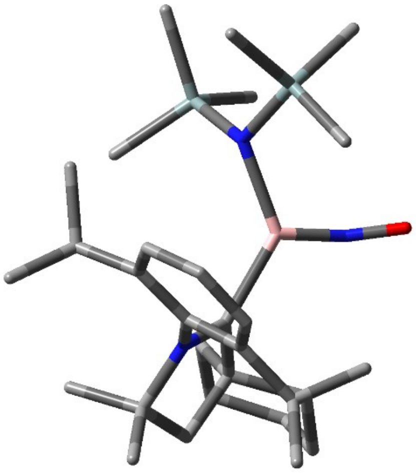

C	2.03756000	1.40243300	1.41638900
C	3.15370500	0.54457500	0.81759600
C	2.48350600	-0.70731100	0.21008400
C	1.00375200	-0.27763700	0.06163200
N	0.86269000	0.97491300	0.59662000
H	3.66060500	1.11220200	0.03126600
C	2.61766700	-1.94209900	1.13737200
C	4.05663600	-2.43677800	1.29378400
C	4.70835000	-2.70140000	-0.06293600
C	4.61582700	-1.47020300	-0.96331500
C	3.16019900	-1.03246000	-1.14497100
H	5.75217100	-3.00102900	0.06882500
H	4.65550700	-1.70341300	1.84667800
H	4.05691000	-3.34829500	1.89886500
H	2.01342900	-2.75266800	0.72042700
H	2.18388200	-1.72239400	2.11573100
H	5.20460700	-0.65293200	-0.52887000
H	5.05473800	-1.68116900	-1.94279700
H	3.10403000	-0.16070000	-1.80216000
H	2.63449800	-1.83802700	-1.65882400
H	4.19593000	-3.54002200	-0.55119900
H	3.90681200	0.29874000	1.56682900
C	2.33851000	2.89323000	1.29157600
H	1.51781200	3.50437900	1.67378500
H	3.23385000	3.11958600	1.87618400
H	2.52725000	3.18248000	0.25765300

C	1.82859100	1.06532000	2.90016700
H	2.76409300	1.22733600	3.44292000
H	1.06843600	1.70156500	3.35170900
H	1.53134900	0.02562700	3.04162300
B	-0.09314500	-1.15780900	-0.45702400
C	-1.92555200	3.45562300	0.46363500
C	-1.13567300	2.39797800	0.91402400
C	-0.06909600	1.96187700	0.11631300
C	0.19372100	2.58577500	-1.12353600
C	-0.62946700	3.62957200	-1.53428100
C	-1.68012700	4.07118500	-0.74791200
H	-2.75237500	3.79708300	1.07698400
H	-0.44362800	4.10938000	-2.48839500
H	-2.30715400	4.88937600	-1.08348100
C	1.33493300	2.16502300	-2.03434400
C	-1.47930900	1.76572800	2.24544100
H	2.01734700	1.55637300	-1.44450100
H	-0.77346200	0.95540100	2.41499000
C	-2.87917900	1.15056200	2.20006800
H	-2.97252300	0.50997900	1.32349200
H	-3.06531900	0.54929400	3.09457900
H	-3.65345800	1.92222500	2.14677700
C	-1.35915100	2.76558300	3.40104900
H	-1.49611800	2.25889500	4.36078900
H	-0.38527600	3.26196200	3.41167700
H	-2.12200300	3.54608100	3.32622300
C	2.14367600	3.34999100	-2.57426900
H	2.45094400	4.03282200	-1.77806000
H	3.04337700	2.98456700	-3.07725000
H	1.57630400	3.92937100	-3.30770300
C	0.82329800	1.29458400	-3.18701700

H	0.32325400	0.40519400	-2.80227600
H	0.11495500	1.85113300	-3.80866200
H	1.65305200	0.97250300	-3.82379800
N	-1.59384100	-1.22757900	-0.24962600
Si	-1.96480300	-2.30758900	1.09115000
Si	-2.63564900	-0.92412500	-1.62917500
C	-3.75575500	-2.26373000	1.68470300
H	-4.40899300	-2.87284900	1.05766500
H	-3.77215600	-2.70211000	2.68875600
H	-4.18922200	-1.26558600	1.75736200
C	-1.66667000	-4.11190600	0.62754100
H	-2.01417700	-4.76312800	1.43688000
H	-2.22119900	-4.38395200	-0.27663900
H	-0.61236000	-4.33950000	0.45241900
C	-0.86146700	-1.89104800	2.56664200
H	-0.30170000	-0.97454700	2.38514200
H	-1.45225900	-1.75415100	3.47708800
H	-0.13449000	-2.68641100	2.75340700
C	-4.33846500	-1.70137000	-1.43110100
H	-4.29434500	-2.78332700	-1.27793900
H	-4.91961100	-1.26307000	-0.61721500
H	-4.88870800	-1.52601100	-2.36166900
C	-1.92608700	-1.67327400	-3.20667700
H	-1.82304800	-2.75929700	-3.12192700
H	-2.60786600	-1.46813100	-4.03862200
H	-0.94822000	-1.26693600	-3.47596700
C	-2.89993900	0.90568200	-1.91299900
H	-3.31593900	1.38960100	-1.02565300
H	-1.96799700	1.41710900	-2.15572500
H	-3.59702500	1.06015400	-2.74335400
C	0.40422400	-2.36303000	-1.16916800

O	0.67453700	-3.31923900	-1.75105900

NCB-PH_3 (6)

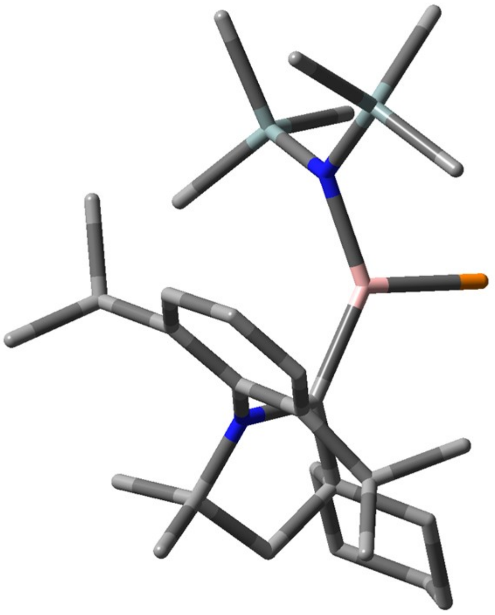

C	1.86765300	1.42011200	1.56473500
C	3.07790500	0.70006300	0.94516700
C	2.54647600	-0.56868700	0.24040100
C	1.03366200	-0.26996400	0.06844900
N	0.76147000	0.97300900	0.69082400
H	3.55220100	1.36314000	0.21497200
C	2.76493100	-1.82072100	1.12950000
C	4.23357900	-2.20651100	1.32435800
C	4.96005700	-2.35957800	-0.01048100
C	4.79769600	-1.10148700	-0.86214700
C	3.31755400	-0.77951600	-1.08194000
H	6.01922100	-2.57933700	0.15320700
H	4.74621700	-1.44676100	1.92561000
H	4.28988500	-3.13749000	1.89690000
H	2.23532200	-2.66668300	0.67956200

H	2.29586200	-1.66372800	2.10265100
H	5.29188600	-0.25725700	-0.36657700
H	5.29331300	-1.22812400	-1.82966900
H	3.20822000	0.09838300	-1.72146700
H	2.89410100	-1.61801400	-1.64368100
H	4.54000700	-3.21670600	-0.55431800
H	3.83478700	0.47698400	1.69917400
C	2.06797800	2.93421900	1.54879600
H	1.18549600	3.46098800	1.91675600
H	2.91081800	3.19113100	2.19609000
H	2.28503100	3.30194200	0.54556100
C	1.65740300	0.96980500	3.02150900
H	2.57976100	1.10599900	3.59446200
H	0.87484500	1.55341000	3.50802500
H	1.37056700	-0.08045500	3.07496600
B	-0.01711700	-1.12809300	-0.46697600
C	-2.21280400	3.17277000	0.24290700
C	-1.32495200	2.26282900	0.81540900
C	-0.16528500	1.89337900	0.11024500
C	0.09604700	2.47532400	-1.15254600
C	-0.84443800	3.33904000	-1.70702700
C	-1.99502000	3.69135000	-1.02001000
H	-3.10285800	3.46328400	0.79154200
H	-0.65607100	3.77338500	-2.68346800
H	-2.70762300	4.37948900	-1.46095300
C	1.40830300	2.27249800	-1.89315100
C	-1.62968700	1.71912200	2.19641500
H	2.07648300	1.75262300	-1.21199700
H	-0.88659500	0.94724900	2.39111100
C	-3.01065200	1.06530700	2.27453000
H	-3.11039000	0.29023900	1.51708400

H	-3.16134600	0.60923600	3.25796000
H	-3.81422700	1.79213900	2.12131700
C	-1.50203000	2.80910200	3.26755700
H	-1.63456700	2.38173500	4.26643400
H	-0.52813200	3.30239500	3.23599100
H	-2.26567900	3.58131200	3.13013800
C	2.07247300	3.61023200	-2.24802100
H	2.11366000	4.28493500	-1.38958800
H	3.09418700	3.43960100	-2.60056800
H	1.53403400	4.12749000	-3.04750200
C	1.27485400	1.40538900	-3.14707900
H	0.92385300	0.40744700	-2.88434400
H	0.56892300	1.84306000	-3.85978400
H	2.24367200	1.31339000	-3.64883800
N	-1.49586600	-1.24241000	-0.25456000
Si	-1.85330100	-2.32815600	1.08120900
Si	-2.66969500	-0.91078200	-1.51547800
C	-3.70563200	-2.40549700	1.47172100
H	-4.08902700	-3.41845100	1.31617900
H	-3.87378000	-2.14764900	2.52130500
H	-4.32093100	-1.73239100	0.87449600
C	-1.33740900	-4.10193900	0.66695100
H	-1.66019200	-4.76892200	1.47337700
H	-1.80569100	-4.45744700	-0.25645800
H	-0.25244900	-4.21760500	0.57616200
C	-0.94503500	-1.89538500	2.66181900
H	0.12614000	-1.81011900	2.47682000
H	-1.29478600	-0.96330100	3.10738200
H	-1.10513000	-2.69633200	3.39190400
C	-3.51871400	-2.51349700	-2.05348000
H	-2.80586500	-3.22819300	-2.47847300

H	-4.03274400	-3.01285500	-1.22761000
H	-4.26629200	-2.30174800	-2.82484200
C	-1.83627500	-0.18297300	-3.02853400
H	-1.06223200	-0.81757700	-3.46951000
H	-2.59713900	-0.01577800	-3.79822000
H	-1.38788200	0.78134600	-2.78399800
C	-4.01774300	0.31495000	-1.05878200
H	-4.66349400	-0.00070300	-0.23713300
H	-3.57986400	1.27970100	-0.79577800
H	-4.65422700	0.46405800	-1.93800700
P	0.59063400	-2.55774800	-1.68205000
H	1.50502700	-3.58296700	-1.33691400
H	-0.50337700	-3.33519300	-2.09463200
H	1.13248600	-2.22509800	-2.94489800

NCB(H)-C_6H_5 (7)

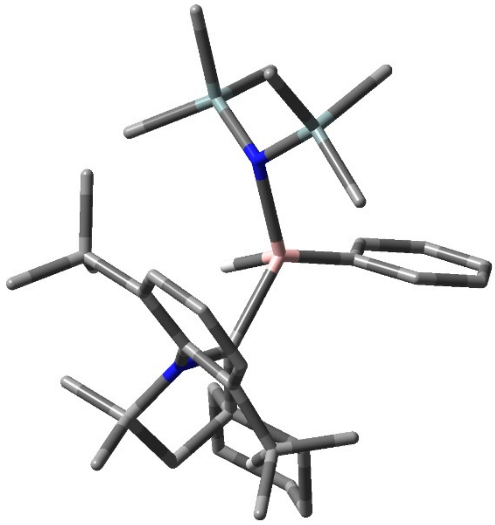

C	-2.05724433	2.07694114	1.00454086
C	-1.38251133	3.02101514	0.02119686

C	-0.02294233	2.34482914	-0.29825014
C	-0.29799533	0.83107214	0.03420186
N	-1.53850733	0.75845614	0.46660486
H	-1.99420833	3.12613414	-0.87924414
C	1.10079567	3.16109114	0.40008486
C	1.22198567	4.57384214	-0.19515714
C	1.49574667	4.52700214	-1.69596214
C	0.40507267	3.73695314	-2.41156314
C	0.27309467	2.33481414	-1.81614314
H	1.57468767	5.53797014	-2.10670714
H	0.32802067	5.18122914	-0.01108214
H	2.04691667	5.07834814	0.31815086
H	2.09085267	2.72047014	0.30656486
H	0.90270167	3.26305814	1.47029686
H	-0.55044333	4.27134814	-2.33807214
H	0.62932167	3.64576714	-3.47833914
H	-0.50066433	1.77730914	-2.34526414
H	1.21669467	1.80544514	-1.96824814
H	2.46116467	4.03591614	-1.86967814
H	-1.27122533	4.01486614	0.45001586
C	-3.57509333	2.19081914	1.04550886
H	-4.01198533	1.43666114	1.70325086
H	-3.82288433	3.17536814	1.45059986
H	-4.04100533	2.10584414	0.06484486
C	-1.50957633	2.26320814	2.43301886
H	-1.49517733	3.32779614	2.67767686
H	-2.13867333	1.75562914	3.16593086
H	-0.49245933	1.87987214	2.53280686
B	0.85751367	-0.43588886	0.29005186
C	-3.49858933	-2.27390686	1.47070886
C	-2.53038033	-1.26909086	1.54529186

C	-2.42227033	-0.39127886	0.46190686
C	-3.30084133	-0.47084986	-0.64303014
C	-4.23345033	-1.49925286	-0.66784114
C	-4.33339533	-2.40037586	0.37993886
H	-3.59471933	-2.97073186	2.29533086
H	-4.89997833	-1.59746386	-1.51474914
H	-5.07397833	-3.19113786	0.34928086
C	-3.21441333	0.47406214	-1.83821314
C	-1.68315033	-1.19991886	2.80250286
H	-2.86148233	1.43828014	-1.47582414
H	-0.98323733	-0.37277786	2.69554586
C	-0.86975733	-2.48738086	2.97568686
H	-0.25096133	-2.67212786	2.09695286
H	-0.22010833	-2.41347686	3.85214386
H	-1.52643233	-3.35026986	3.11980486
C	-2.54782333	-0.93191686	4.04145786
H	-1.91091733	-0.77292786	4.91598686
H	-3.17641533	-0.04643286	3.91254986
H	-3.20853933	-1.77510086	4.26164586
C	-4.56611733	0.73140114	-2.50885314
H	-5.33181033	1.01928914	-1.78319014
H	-4.46471733	1.53878114	-3.23865714
H	-4.92678633	-0.14607086	-3.05191014
C	-2.18902833	-0.00391986	-2.86211014
H	-1.21471633	-0.13311086	-2.39316714
H	-2.48167333	-0.96060886	-3.29972714
H	-2.08526233	0.72853614	-3.66832814
N	1.33066067	-1.70040886	-0.54156714
Si	2.57960767	-2.65258986	0.26216886
Si	0.68240467	-2.27221586	-2.04658314
C	2.16015167	-4.49963686	0.26411586

H	2.34743267	-4.99639086	-0.68619414
H	2.77928467	-4.99962286	1.01638586
H	1.11270667	-4.66775486	0.53597386
C	4.24737667	-2.40451086	-0.58240814
H	5.00082367	-3.09179386	-0.18226614
H	4.19145367	-2.56185686	-1.66289514
H	4.59712467	-1.38237986	-0.41298614
C	2.86348567	-2.25346086	2.08234786
H	3.57349367	-1.43355486	2.20148486
H	1.95203967	-1.99511086	2.62052986
H	3.29285567	-3.14191486	2.55719586
C	1.54700967	-3.78216786	-2.79859314
H	2.63613367	-3.78479086	-2.71939214
H	1.17019767	-4.71978986	-2.38734614
H	1.30332567	-3.77638186	-3.86759114
C	0.94841867	-0.98505886	-3.40349214
H	2.00286367	-1.06374986	-3.69159414
H	0.34987067	-1.19777186	-4.29570614
H	0.77086167	0.04661914	-3.11068314
C	-1.06527533	-2.93309686	-1.80775914
H	-0.95666233	-3.79685286	-1.14236014
H	-1.76205333	-2.25257286	-1.33044414
H	-1.51201433	-3.28871586	-2.74271114
H	1.11660544	-0.41881361	1.48200963
C	2.18803802	0.51132682	0.86801695
C	3.10871402	0.96815382	-0.08998505
C	2.32432202	0.98102082	2.18097995
C	4.08633802	1.89173782	0.27897695
H	3.06816402	0.57186482	-1.10089805
C	3.31321602	1.88310782	2.54602295
H	1.63216702	0.61502082	2.93505395

C	4.18997902	2.35794582	1.58667395
H	4.78119402	2.25481782	-0.47287805
H	3.39197002	2.22546982	3.57299795
H	4.95273502	3.08264582	1.84877595

NCB(H)-CH_3 (**8**)

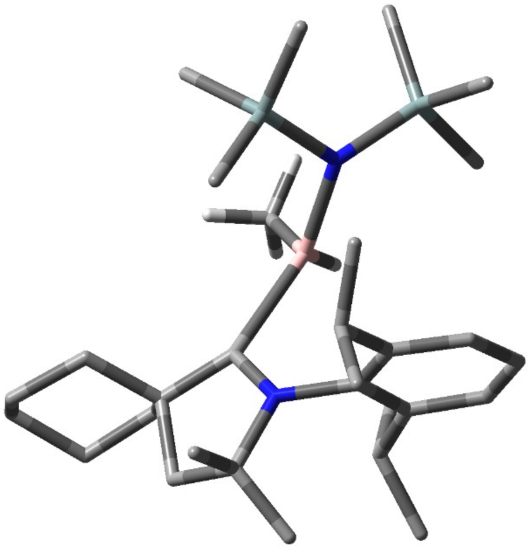

C	2.18992100	1.27971200	1.38378200
C	3.25156700	0.44384100	0.67539100
C	2.52972000	-0.74507300	0.01041600
C	1.09105900	-0.25280900	-0.08833200
N	0.95703900	0.89583900	0.56307300
H	3.73323400	1.05240700	-0.09863800
C	2.60408200	-2.05526500	0.83147900
C	4.01924000	-2.61190400	0.93105700
C	4.67133100	-2.78111600	-0.43296300
C	4.61119100	-1.48460000	-1.23002100
C	3.16606800	-1.01730800	-1.37469100

H	5.70486100	-3.11985900	-0.32284200
H	4.63207300	-1.94324300	1.54901900
H	3.99114400	-3.56446600	1.46801200
H	1.94753300	-2.79722500	0.35311900
H	2.20056400	-1.90317900	1.83509800
H	5.21835300	-0.71193500	-0.74109400
H	5.04481200	-1.62153800	-2.22457200
H	3.09823500	-0.11707900	-1.99820100
H	2.62203200	-1.80872200	-1.89262200
H	4.14907800	-3.56941400	-0.99236500
H	4.03661900	0.13368200	1.36704100
C	-1.17572500	2.04418600	1.15557000
C	-0.11002200	1.84168700	0.26510700
C	0.00292400	2.57411000	-0.94075300
C	-1.02628400	3.46449400	-1.25162000
C	-2.10796500	3.65033900	-0.40669400
C	-2.16649400	2.95767800	0.78993800
H	-0.96394200	4.03409900	-2.17406300
H	-2.89316500	4.35025000	-0.67229000
H	-2.99960200	3.12435200	1.46598300
C	1.19463800	2.52465400	-1.88648900
C	-1.29648300	1.36360500	2.50081600
H	1.96075700	1.86890900	-1.45542900
H	-0.50648700	0.61174000	2.57532100
C	0.84106600	1.98078000	-3.27124800
H	0.06401000	2.58654500	-3.74704400
H	1.71756000	2.00445000	-3.92453100
H	0.47300700	0.95310200	-3.24607800
C	1.81067400	3.91895600	-2.05552600
H	2.76187300	3.85588600	-2.59029500
H	1.15471400	4.56682200	-2.64285400

H	1.98726600	4.42297000	-1.10339100
C	-1.11798200	2.37897300	3.63215600
H	-1.94435300	3.09563100	3.64388300
H	-1.10334600	1.88011600	4.60515600
H	-0.19628800	2.95901600	3.53383800
C	-2.63010000	0.63888900	2.64885900
H	-3.47509400	1.33325100	2.63234000
H	-2.77506200	-0.07437400	1.83741300
H	-2.67679600	0.10028800	3.59976100
C	2.46211200	2.76966700	1.32317900
H	1.63106900	3.35441300	1.72603800
H	3.34291700	2.98924500	1.93091400
H	2.66991900	3.10949500	0.30925700
C	2.02166500	0.85240300	2.84079500
H	2.98597000	0.96436000	3.34305400
H	1.30617500	1.47552500	3.37577100
H	1.71700600	-0.19046000	2.94389100
B	-0.13333300	-0.87839700	-0.85905400
N	-1.44783600	-1.21427100	-0.37851800
Si	-1.59311900	-2.50751200	0.85291300
Si	-2.85478100	-0.90180500	-1.45398000
C	-0.58327700	-2.10712400	2.37992100
H	0.22037100	-1.40371800	2.16446900
H	-1.19943200	-1.66569700	3.16652300
H	-0.12451000	-3.01249300	2.78832900
C	-0.93888600	-4.09466600	0.09234300
H	-1.41017600	-4.32755300	-0.86716800
H	0.14448000	-4.08027200	-0.06457800
H	-1.13987400	-4.93824300	0.76034700
C	-3.35958600	-2.81818800	1.38717900
H	-4.05957400	-3.03661200	0.57936000

H	-3.33646500	-3.70782800	2.02636500
H	-3.78302400	-2.01337200	1.99081100
C	-4.21593500	-0.04843900	-0.50313500
H	-4.61295700	-0.62143800	0.33605100
H	-3.87721700	0.91921100	-0.12316200
H	-5.05041400	0.14353700	-1.18583200
C	-3.49102400	-2.48611200	-2.23650400
H	-2.76908100	-2.93780300	-2.92373500
H	-3.78639200	-3.26241400	-1.52741900
H	-4.37869800	-2.25271200	-2.83361400
C	-2.35087900	0.24865700	-2.84327500
H	-1.89657200	1.17387100	-2.47790300
H	-1.68079300	-0.19354500	-3.58629500
H	-3.25925500	0.53411400	-3.38429900
C	-0.03342619	-2.09666560	-2.17882183
H	-1.00001819	-2.41452060	-2.55510483
H	0.03269400	-0.50623600	-2.04636300
H	0.43012281	-2.88111060	-1.57732883
H	0.63383081	-1.79811660	-2.98735783

NCB(H)-(H) (**9**)

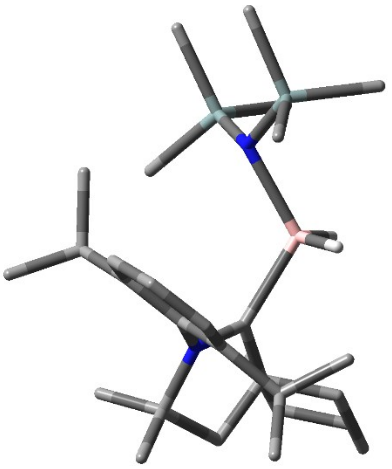

C	2.03733800	1.31983600	1.48199100
C	3.15016100	0.45318000	0.88798800
C	2.46599700	-0.77280900	0.24415100
C	0.99321900	-0.31190100	0.08936400
N	0.88023900	0.94672900	0.61566300
H	3.68287600	1.02779200	0.12412700
C	2.58734100	-2.02758800	1.14512000
C	4.01805600	-2.55399100	1.27686000
C	4.65560200	-2.80109600	-0.08913400
C	4.58594800	-1.54433000	-0.95474600
C	3.13884100	-1.07432800	-1.11938100
H	5.69266700	-3.12849200	0.02931200
H	4.63470100	-1.84315900	1.83972300
H	4.00486200	-3.47752500	1.86313500
H	1.95972500	-2.82051100	0.72933600
H	2.18307800	-1.81195600	2.13714300
H	5.19127400	-0.75109500	-0.49865500
H	5.01821400	-1.73562700	-1.94122400
H	3.09825900	-0.18431700	-1.75197200
H	2.59573900	-1.85416800	-1.65480300
H	4.12094100	-3.61461900	-0.59564000
H	3.88353900	0.17704400	1.64624800
C	2.37919900	2.80519700	1.41808100
H	1.55624100	3.42447700	1.78189600
H	3.25078200	2.99094800	2.05090800
H	2.62319500	3.12255800	0.40362500
C	1.76262100	0.93452100	2.94404700
H	2.68916300	0.99885500	3.52141500

H	1.03833500	1.60703500	3.40206500
H	1.37436700	-0.08155200	3.02507400
B	-0.11950800	-1.16353300	-0.43951400
C	-1.84310400	3.49561700	0.44887600
C	-1.09393400	2.40905000	0.89932300
C	-0.01200700	1.96490600	0.12781600
C	0.30464200	2.60869200	-1.08866700
C	-0.48089200	3.67976100	-1.50319000
C	-1.54502600	4.13058500	-0.74046600
H	-2.67995800	3.84439300	1.04411500
H	-0.25341300	4.17426800	-2.44060100
H	-2.14069400	4.97138500	-1.07725200
C	1.46427800	2.17784700	-1.96966300
C	-1.49165600	1.75671300	2.20505600
H	2.11946100	1.55438900	-1.36499100
H	-0.82431700	0.90934400	2.35840000
C	-2.92014600	1.21520300	2.12005900
H	-3.02195800	0.57793500	1.24235700
H	-3.16650400	0.62698400	3.00895200
H	-3.65140100	2.02577300	2.04559300
C	-1.35108100	2.72161900	3.38850200
H	-1.51153700	2.19536500	4.33410300
H	-0.36442100	3.19046900	3.42074000
H	-2.09121200	3.52486800	3.32629700
C	2.30800200	3.35368900	-2.47387900
H	2.60430400	4.02120600	-1.66055500
H	3.21506000	2.97790000	-2.95556600
H	1.77177500	3.95111400	-3.21622500
C	0.97114600	1.32385600	-3.14259700
H	0.45061900	0.43717500	-2.77850900
H	0.28464100	1.89456200	-3.77575400

H	1.81213000	0.99721100	-3.76215400
N	-1.62626300	-1.18449500	-0.26844800
Si	-2.08763100	-2.26045200	1.04661600
Si	-2.60798800	-0.83812200	-1.68311800
C	-3.90192200	-2.16839500	1.55808200
H	-4.53803500	-2.79666600	0.93197800
H	-3.97021000	-2.55480000	2.58107200
H	-4.32047900	-1.16122700	1.56214700
C	-1.79321100	-4.06621300	0.58550800
H	-2.21063900	-4.72044100	1.35815000
H	-2.28532700	-4.31489100	-0.36051900
H	-0.73353100	-4.31272700	0.48165700
C	-1.08446300	-1.91260800	2.59914500
H	-0.01534500	-1.86327000	2.38903000
H	-1.38671300	-0.98232000	3.08451300
H	-1.24905100	-2.72598700	3.31379400
C	-4.33355900	-1.58199000	-1.56850100
H	-4.31924300	-2.66536200	-1.41974000
H	-4.94514700	-1.13598400	-0.78176200
H	-4.83509100	-1.39134800	-2.52330300
C	-1.85365700	-1.58066100	-3.24307300
H	-1.78588400	-2.67069400	-3.17519300
H	-2.49446600	-1.34078200	-4.09803500
H	-0.85434700	-1.19874600	-3.46461400
C	-2.82260000	1.00016100	-1.95413100
H	-3.27932000	1.48164300	-1.08574400
H	-1.86909800	1.49650100	-2.13792100
H	-3.46807800	1.17815100	-2.82073900
H	0.03028184	-1.05540173	-1.78387989
H	0.24621422	-2.11183151	-0.78378250
